# Particle‐Based Detection of Surface Chemistry via Optical Microscopy—Integrating Microfluidics, Light‐Induced Activity of Colloids and Data Science

**DOI:** 10.1002/smtd.202502329

**Published:** 2026-02-27

**Authors:** Fabian Rohne, Daniela Vasquez Muñoz, Isabel Meier, Anne Nitschke, Florian Schmitt, Nino Lomadze, Martin Reifarth, Andreas Taubert, Svetlana Santer, Marek Bekir

**Affiliations:** ^1^ Institute of Physics and Astronomy University Potsdam Potsdam Germany; ^2^ Institute of Chemistry University Potsdam Potsdam Germany; ^3^ Institute of Chemistry, Fraunhofer Institute of Applied Polymer Research University Potsdam Potsdam Germany

**Keywords:** colloid sensors, particle‐based porosity determination, photosensitive surfactants, surface area determination, unsupervised machine learning

## Abstract

We present particle‐resolved methods for determining the porosity and surface area of microparticles, based on single‐particle trajectory analysis conducted via optical video microscopy integrated with microfluidics and LED illumination. This technique introduces a unique combination of analytical advantages that address key limitations of conventional methods, such as BET nitrogen adsorption. Notably, (1) the method operates with extremely low analyte quantities on the order of micrograms or less and in the form of dilute aqueous dispersions, eliminating the need for drying or bulk sample preparation; (2) surface area quantification is performed on a per‐particle basis, with total surface area determined by summing up all individual particle measurements, enabling high‐resolution analysis of particle‐to‐particle heterogeneity; and (3) the entire workflow from sample preparation to data acquisition and surface area calculation is rapid, straightforward, and relies only on a pre‐defined, self‐generated reference data library. We validate the method using both plain and mesoporous SiO_2_ microparticles, demonstrating a relative precision of approximately 9%, in line with benchmark techniques such as nitrogen sorption. This approach thus offers a robust, accessible alternative for surface area analysis, utilizing standard optical microscopy equipment available in most laboratory settings, and is particularly well suited for low‐sample‐volume applications in material characterization.

## Graphical abstract

A particle‐resolved method for porosity and surface area analysis of microparticles is presented, based on single‐particle trajectory tracking via optical video microscopy integrated with microfluidics and LED illumination. Operating with minimal analyte and no drying steps, the approach offers per‐particle analysis using widely available laboratory microscopy setups.

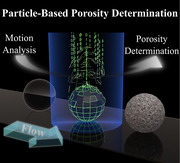

## 1 Introduction

Nanotechnology plays a crucial and growing role in modern science and technology. Many fields, such as medicine, chemistry, electronics, and the automotive industry, use nanomaterials for their unique properties. Because of their tiny size, these materials provide new functions that are changing how we innovate and develop products [1]. Thus, nano‐ and microparticles have become more important for the design and development of modern technologies. In particular, the development of smart and highly responsive materials relies significantly on the ability to modulate surface‐related characteristics. While nanoparticles inherently provide a high surface‐to‐volume ratio due to their size [2], achieving comparable surface area for particles at the submicron or micron scale often necessitates the introduction of a porous morphology. For example, such porous particles based on different bulk materials are used for several different applications, e.g. porous silica particles are used as catalyst supports, adsorbents, molecular sieves, chemical sensors [3, 4, 5, 6], or as medical drug carriers [7], mesoporous TiO_2_ according to high photocatalytic activity are employed in solar energy conversion (e.g., solar cells), metal‐organic frameworks (MOFs) for very high porosity for gas adsorption [8, 9, 10], lithium‐ion batteries, biosensing technologies, and cancer treatment [11, 12], and mesoporous Co_3_O_4_ particles find applications in energy storage and semiconductor industry [13].

Fundamentally, the total porosity (Φ) of a material is commonly defined as the ratio of the pore (void) volume (*V*_p_) to the total volume (*V*_t_) of the material, expressed as Φ = *V*_p_ / *V*_t_. Beyond this quantitative definition, the term *porosity* is often employed more broadly as a general descriptor encompassing a range of related structural characteristics, including pore morphology (e.g., pore volume and pore size distribution) and specific surface area [14, 15, 16].

Given the significant functional roles of mesoporous particles, accurate characterization of their porosity is essential. Although several well‐established techniques are available for this purpose, such as Brunauer‐Emmett‐Teller (BET) and other algorithms for treating gas adsorption data, gas expansion methods, small‐angle X‐ray scattering (SAXS), and electron microscopy (e.g., SEM and TEM), each methods exhibit inherent limitations. These include constraints related to the measurable pore size range, accuracy of the analysis, required sample quantity, and the complexity of sample preparation procedures [17, 18]. Some of the techniques allow for the comparison of the porosity of particles through the determination of some related particle characteristics, such as specific surface area. For instance, the most famous and common method, that is nitrogen sorption, normally requires a high sample amount and provides information about the pore volume and the surface area, *but all the information is averaged over the whole macroscopic sample*; the same applies to several other techniques. Thus, the nitrogen sorption still lacks the quality of analysis with respect to a *particle‐based quantification approach*, in particular for particles on the micron scale. Up to now, there are only two nanoparticle‐based techniques: TEM and focused ion beam SEM (FIB‐SEM). Related reviews [17, 18] and Table 1 from reference [1] give an overview of the commonly used techniques.

**TABLE 1:** Adjusted mass ratio, relative mass ratio with respect to PSiO_2_ *X*_PSiO2_, surface area per mass theoretically calculated from *X*_PSiO2_, surface area per mass from LDV method. (Equation 4).

Mass ratio adjusted		*X*_PSiO2_	BET value theoretically	BET value LDV‐method
PSiO_2_	SiO_2_		m^2^∙g^−1^	m^2^∙g^−1^
0	1	0.00	0.5	0.5
1	4	0.20	170.4	280.9
1	2	0.33	280.9	391.3
2	3	0.40	340.3	314.8
1	1	0.50	425.3	399.8
3	2	0.60	510.2	569.7
2	1	0.66	561.2	646.1
3	1	0.75	637.6	705.6
1	0	1.00	850.0	850.0

Nonetheless, modern applications as mentioned above demand the use of porous particles; ideally, the porosity and apparent surface area are uniformly distributed among all particles; however, this is rarely achieved in practice. And as such, particle‐based porosity determination becomes, with respect to modern technology, urgently demanding since it allows a correlation of physical (form and size) with chemical and interfacial properties (morphology or interface) of the dispersed particles and thus allows a better interpretation of the experimental results and/or better improvement of the material design. Further, it enables characterization of a porosity distribution classified by individual particles and would give valuable information about particle porosity or surface area distribution, too.

**FIGURE 1:**
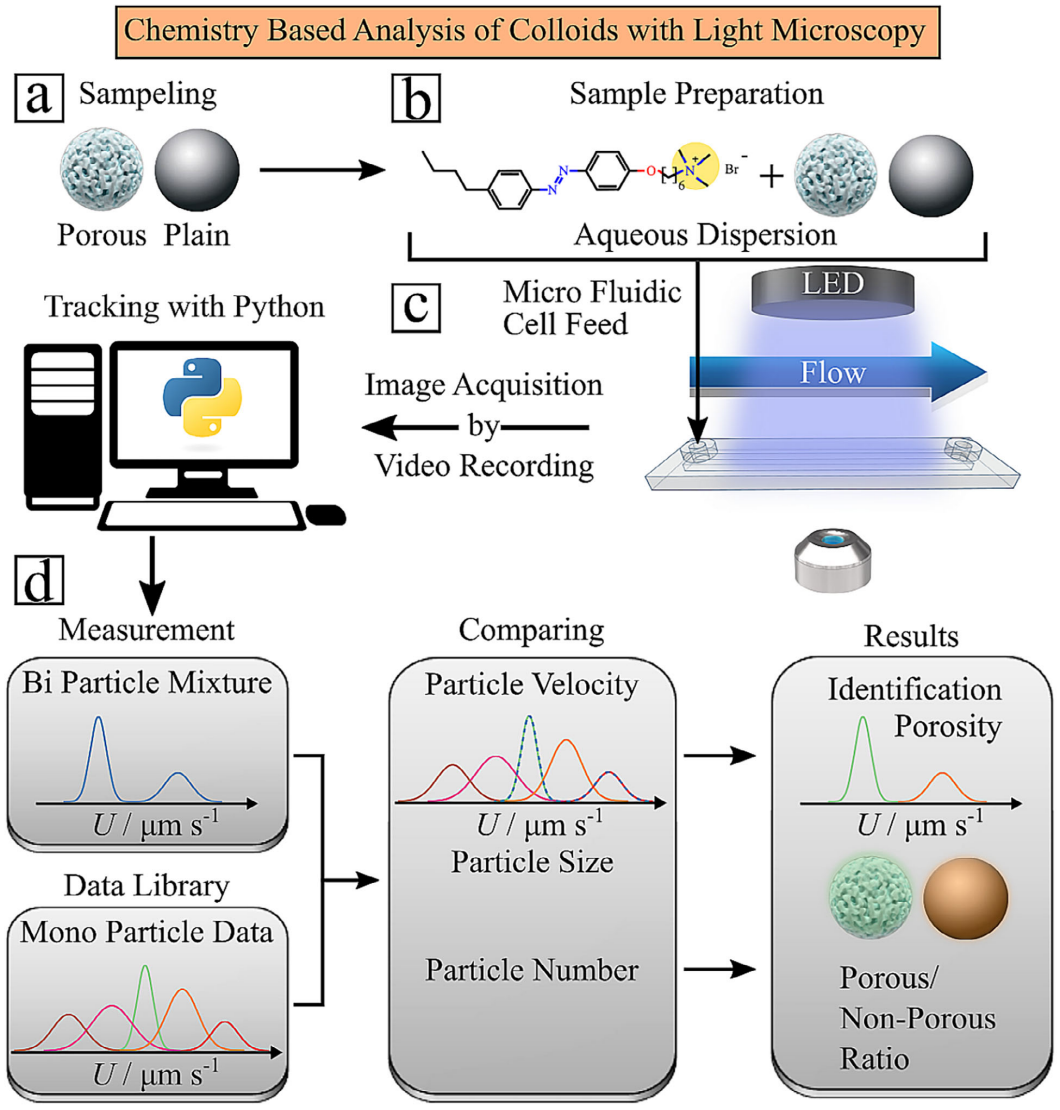
Schematic representation of the surface chemistry‐based analysis via optical microscopy. (a) Microparticles in variation in porosity are dispersed in ionic free water (MilliQ) and (b) mixed with azo‐benzene containing surfactant. This substance renders microsized colloids chemically active upon light illumination. (c) The dispersion is injected into the microfluidic cell. Then particles must sediment at the bottom interface, followed by a switched‐on pressure‐driven fluid flow. Particles crossing the analyzer cell (microfluidic cell) will be illuminated with blue light (455 nm) at fixed intensity and cause the chemically activated particles to hover slightly from the bottom glass substrate. The particles experience a different magnitude of the shear, basically experience a light‐induced drift velocity in the flow, depending on their surface chemistry. (d) The recorded trajectories will be analyzed for each particle, and their average velocity and velocity distribution are compared with a data library to provide qualitative information about surface chemistry. A quantification is achieved by summing up all trajectories giving finally a qualitative and quantitative distribution of dispersed colloids in variation of their surface properties/porosity, thus kind of particle‐based interpretation of the analyte.

In this article, we show a new method towards particle‐based surface area estimation, where the analysis relies on the use of a simple optical microscope in combination with microfluidics and light illumination. The method only requires cheap measurement equipment, which renders such method to be included as standard devices in any laboratory and as a standard daily lab routine task. The probing chamber is built up from a microfluidic channel and contains the analyte (microparticles) dispersed in aqueous suspension with dissolved photosensitive azobenzene‐based surfactant (Figure 1). The latter renders microparticles to be chemically active due to the local‐light‐driven diffusioosmosis (*l*‐LDDO), which relies on a dynamic exchange of molecular isomers occurring at the particle (object) surface. Briefly, this exchange involves the *cis‐* and *trans‐*isomers. The *trans*‐isomer is surface‐active, whereas the *cis‐*isomer exhibits significantly lower surface‐activity. Upon illumination, the two isomers interconvert via photoisomerization. Owing to the different surface activities, *cis‐*isomers of the surfactant are continuously expelled, generating a steady flux of *cis‐*isomers that is proportional to the *trans*‐*cis*‐photoisomerization rate of adsorbed *trans*‐surfactant molecules at the interface of the particles [19]:

(1) $$\mathrm{constant}\;\mathrm{flux}∼\frac{dc_{p}}{dt}=A_{\mathrm{eff}}\cdot \theta_{T}\cdot k_{\mathrm{TC},I}\left(\lambda \right)\cdot I$$

with *A*_eff_ as the apparent surface area of the particle, *I* the light intensity, *k*_TC,I_(*λ*) the photoisomerization rate constant depending on the applied wavelength of illumination *λ*, and *θ*_T_ the *trans*‐isomer surface coverage. The continuous supply of *cis*‐isomers diffusing from the particle interface leads to an accumulation of *cis‐*isomers in the vicinity of the particle surface [19]. This constant flux (Equation 1) induces a local imbalance in the *cis*‐isomer concentration in the vicinity of the particle, which induces *cis*‐isomer concentration gradients along the direction parallel to the interface. This gradient leads the osmotic pressure to squeeze the fluid against the wall; its lateral concentration gradient along a parallel direction at the interface generates an osmotic pressure gradient along the interface. This induces a diffussioosmosis (DO) [20, 21, 22] along the osmotic pressure gradient direction, a kind of “hydrodynamic fluid flow” along solid‐liquid interfaces [22, 23]. In the case of sedimented particles the bottom substrate acts as a reflective boundary where the *cis‐*isomer gradient in the bottom of the particle is high and dissipates vertically along the particle interface and laterally along the bottom substrate. This causes osmotic pressure to laterally and vertically decay, thereby inducing an osmotic and phoretic slip, respectively [24, 25]. Both slips provide a kind of chemical activity and yield an upstream velocity of the particle, causing a levitation of the particle from the bottom wall into the vicinity of the bulk solution up to higher than the diameter of the particles [24, 25]. Since the *l*‐LDDO (i.e., light‐induced chemical activity) depends on the *trans*–*cis*‐isomer flux exchange [19], the magnitude of the upstream velocity is proportional to the degree of chemical conversion of the photosensitive surfactant, as described by Equation (1) [26]. Further details on the physical mechanism underlying levitation can be found in the literature [24] or in the summary provided elsewhere [25].

When levitated particles are exposed to an externally applied laminar flow, the particle experiences shear stress proportional to the levitation height [27, 28]. Height and the resulting light‐induced velocity depend on the magnitude of the flux and therefore on the net amount of adsorbed *trans*‐isomer proportional to the parameters *A*_eff _· *θ*_T_. In the case of equally sized particles composed of the same bulk material as simulated in this work using silica (SiO_2_) microbeads, the mobility of the particles then only varies by the apparent area *A*_eff_. This mobility can be recorded via video microscopy, reversely interpreted into the apparent surface area for one particle and quantified over all particles crossing the optical image area, i.e., into a particle‐based analysis approach. This approach not only provides quantitative information on the total surface area but also insights into the distribution of surface areas across the distribution of individual particles. In this work, we present and elucidate the underlying principles of the analytical method, detailing the procedures for data acquisition, processing, and interpretation. The analysis leverages readily accessible unsupervised machine learning algorithms [29, 30, 31], demonstrating how such tools can be effectively applied to extract linear‐particle trajectories from optical microscopy data.

## 2 Results and Discussion

### 2.1 Principles of Measurements

The method of detection is illustrated in Figure 1. The required hardware includes an integrated microfluidic cell (analyzer) mounted on an optical microscope with video recording capability, a pump system, and a light source providing the analyzer with a homogenous intensity profile across the entire microfluidic cell. The analyte, containing microparticles as a single dispersion or as a mixture of particles with varying interfacial properties (Figure 1a), is mixed with an aqueous solution of the photosensitive surfactant (Figure 1b) at a concentration of 2 mM for all samples. After an equilibration time, the particle dispersion is injected into the fluid cell. Before the measurement, particles are allowed to sediment undisturbed to the bottom of the cell. Once settled, flow aligns them with the fluid streamlines. Upon light illumination, particles become chemically active, causing vertical levitation toward regions of higher shear. This activity increases their drift velocity, which scales with interfacial properties such as porosity. This *light‐induced drift velocity* (LDV) is the key mechanism enabling particle‐based chemical analysis via optical microscopy in combination with microfluidics. The method starts with framewise recording the LDV of the particles using optical image acquisition. For each particle that crosses the field of view of the attached camera, its trajectory is tracked, and the corresponding velocity is calculated. Thereby, the LDV distributions, being characteristic for the particles’ interfacial properties, are measured as illustrated in Figure 1c, that is, variation in porosity leads to variations in LDV. In a binary mixture of porous and plain particles, differences in velocity are uniquely related to their surface area: plain particles exhibit slower LDV, while porous particles show faster LDV.

The LDV depends on multiple parameters, including that of fluid dynamics (e.g., cell geometry, flow rate) [19, 32], light illumination (e.g., applied wavelength, intensity, illumination pattern) [19, 32] and the physical and chemical properties of the particles (e.g., size, size distribution and surface chemistry) [19, 32]. In actual measurements, parameters related to fluid dynamics and illumination are precisely controlled and kept constant so that variations in LDV arise merely from particle properties. Since optical video microscopy simultaneously measures both particle size and velocity, size information is inherently available. Thus, the only unknown parameter influencing the LDV is the surface chemistry of the particles. The LDV readout can therefore be used to infer surface chemistry (e.g., porosity) of colloids. The method makes use of this principle by comparing the LDV of a particle of known size with a pre‐categorized data library of particles possessing the same size but a varying surface chemistry, as illustrated in Figure 1d. A simple example is shown in Video S1, where blue light illumination at 470 nm and an intensity of 11.5 mW cm^−2^ is applied to a mixture of plain and porous silica microparticles of equal size (*D* = 5 µm). For all measurements, whether single dispersion or mixture, all parameters (flow rate, analyzer geometry, wavelength, and intensity) are kept constant, so that differences arise solely from particle properties. As soon as the illumination is switched on (after 5 s), the velocities of plain and porous particles in single dispersions match those in the binary mixture. Video S1 illustrates this by showing overlapping average velocities and velocity distributions between the single dispersion (model library) and the binary mixture (model analyte). The matching of LDV will be used to design a new optical sensor for colloids that is sensitive to surface properties. The analysis is automated using unsupervised machine learning algorithms, specifically Gaussian mixture model classification. These clustering algorithms are designed to identify natural groupings in data such that items within the same cluster are more similar to each other than to those in different clusters [29]. We chose an unsupervised learning approach, since the particle composition of the analyte is unknown and may vary from sample to sample, making it difficult to obtain reliable training data. In the subsequent step, the velocity distribution of each individual cluster is compared with the velocity distributions stored in the library. This comparison can be performed either manually using violin plots or fully automatically using quantile–quantile (Q–Q) plots. In the next section, the principles of analysis are described in more detail.

### 2.2 Details of Data Recording

**FIGURE 2:**
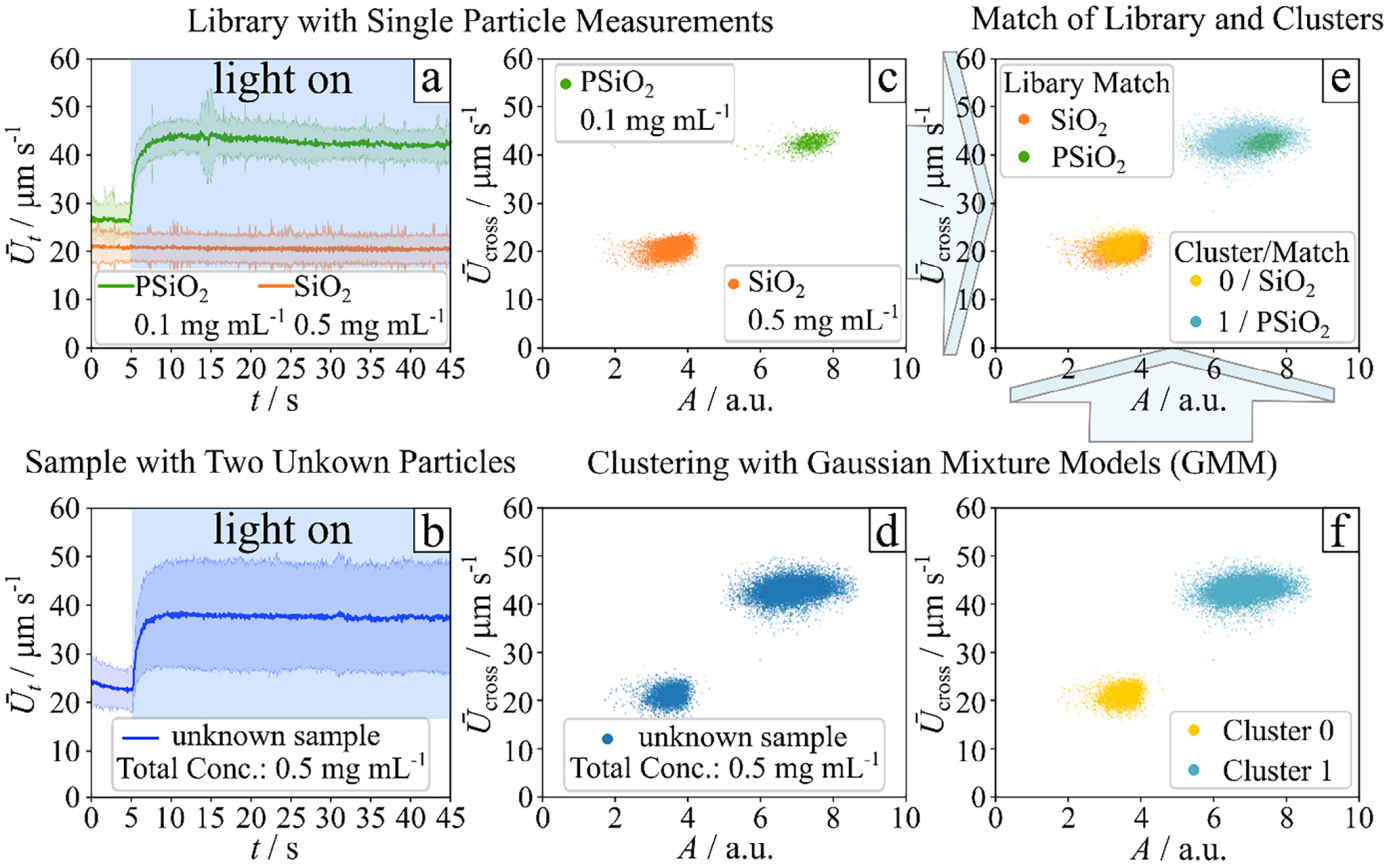
(a,b) example data display style, where the average velocity of the particles is plotted as a function of time series. (a) model library measured for individual dispersed particles and (b) measurement sample containing a binary particle mixture of plain and porous particles (number ratio 2 to 1). The area emphasized in blue illustrates the illumination range with blue light (455 nm). (c,d) Example data display style, which is used to classify the particles in distinct clusters. The average velocity is shown as a function of the recorded particle sizes in pixel area. Each data point corresponds to one trajectory where the particle is crossing the detection area. (c) library data, with a priority classification from a single dispersion collection. (d) Measurement data, where data points are not classified yet. (f) Same measurement data as in d) but clustered with the Gaussian mixture model. (e) overlay of the data in (f) clustered measurement data and in (c) a priori classified library data to provide data interpretation of measured tracks for particle surface chemistry classification. The following sample sizes were determined: library data PSiO_2_ *n*_sample_ = 1217, library data SiO_2_ *n*_sample_ = 6734, analyte data *n*_sample_ = 20833.

To demonstrate the particle‐based detection in more detail, we first prepared the samples by mixing quasi equallysized porous [PSiO_2_, *D* = (5 ± 1.0) µm] and plain [SiO_2_, *D* = (5 ± 0.24) µm] silica particles with an azobenzene‐containing surfactant solution, similar as discussed in the previous section. We adjusted the surfactant concentration to 2 mM (2·10^−3^ mol·L^−1^), and the overall particle mass concentration to 0.5 mg·mL^−1^. Immediately after the preparation, the samples were left to equilibrate for 24 h or longer and stored in the dark to prevent undesired photoisomerization. In total, we prepared three samples: two single dispersions, composed of one with plain SiO_2_ and one with porous PSiO_2_ particles, and one binary mixture with a 1:1 number ratio of SiO_2_ and PSiO_2_ particles. We measured the LDV under blue light illumination (*λ* = 455 nm) at an intensity of 14.3 mW·cm^−2^, and set a flow rate of 150 µL·min^−1^. The velocities of all particles crossing the detection area (entire image, see Video S2) were tracked. The average velocity of all crossing particles is shown in Figure 2a for the single dispersions and in Figure 2b for the binary mixture. The bold line represents the mean velocity, while the transparent area indicates the standard deviation of the velocity. When the light is switched off (*t* = 0 s), particles in both the single dispersion and the binary mixture move at a similar speed. However, upon switching on the blue light (*t* = 5 s), both SiO_2_ and PSiO_2_ particles become chemically active, exhibiting different LDVs due to differences in surface ‐ plain SiO_2_ (BET = 0.54 m^2^·g^−1^) versus porous PSiO_2_ (BET = 850 m^2^·g^−1^). Due to its smaller surface area, SiO_2_ shows a relatively small LDV of (23 ± 3) µm·s^−1^, comparable to the velocity without illumination and consistent with literature values at the given flow rate and light intensity [24]. In contrast, PSiO_2_, with its higher porosity and accordingly surface area, exhibits a significant velocity increase from (27 ± 3) to (44 ± 3) µm·s^−1^, in good agreement with our previous findings [24]. The measured average LDV in the binary mixture was (38 ± 12) µm·s^−1^, which lies between the values observed for pure PSiO_2_ and SiO_2_ dispersions.

However, the time dependency of the mean frame‐to‐frame particle velocity, *U*_t_, is not suited for particle identification, as each finite time *t* represents an ensemble average over all trajectories. This averaging process inherently obscures information about individual particle dynamics. Therefore, an alternative classification scheme is required. Our proposed approach is based on the fundamental physical principles of conservation of mass and momentum.

From basic fluid dynamics, one can derive an analytic expression for levitating particles, giving their velocity as a function of size and lifting height:

(2) $$U_{\mathrm{LDV}}\left(a,h_{\mathrm{lev}}\right)=S\cdot \left[\left(a+h_{\mathrm{lev}}\right)-\frac{5}{16}\cdot \frac{a^{3}}{{\left(a+h_{\mathrm{lev}}\right)}^{2}\;}\right]$$

With *U*_LDV_(*a*, *h*_lev_) the LDV proportional to the particle radius *a*, axial vertical lift of distance of the particle from the bottom interface *h*_lev_ at switched on activity, and the applied shear rate *S*. Since *S* is constant and determined by the set flow rate and cell geometry, in equation (2), the remaining free variables are the levitation distance and the particle size. These two variables are crucial when comparing *U*_LDV_ values from the reference library with those of the analyte. Thus, instead of plotting the average velocity over each finite time interval, it is important to classify the velocity with respect to the particle size. Accordingly, we calculate the average velocity over the entire time a particle takes to cross the optical image and plot the average crossing velocity *U*_cross_ as a function of the pixel area of the tracked particle. Since particles exhibit a rapid LDV burst in the initial time of light exposure, we only consider velocities in the steady‐state non‐equilibrium activity of the particles. This corresponds to the time window from 10 to 45 seconds, as shown in Figure 2c for single dispersion and in Figure 2d for the mixture. Each data point in Figure 2c,d represents one trajectory and contributes to clusters of velocity values. The velocity at the center of a cluster is proportional to the value of *U*_t,ave_ for SiO_2_ and PSiO_2_ (compare Figure 2a with Figure 2c). It is important to mention that *U*_cross_ values include outliers. These outliers influence the cluster identification and thus must be removed before the clustering algorithm is applied. Details on outlier removal and the reason for their appearance are discussed in Section 4.3.

While the single dispersions are independent measurements and therefore are separate datasets of samples, where all the particles possess the same surface features, this is not the case for data sets recorded from a mixture. Let us therefore examine the example shown in Figure 2e, where two distinct clusters can be observed: one with particles exhibiting lower LDVs and another with higher LDVs. However, what is obvious to a human observer must also be recognized by the software using an unbiased algorithm. This is where machine learning, or more specifically, clustering analysis, comes into play. Such algorithms identify natural groupings in data, ensuring that items within the same cluster are more similar to each other than to those in a different cluster [29]. We chose the Gaussian Mixture Model as our clustering algorithm, because the velocity distribution of the majority of our measurements follows a classical Gaussian profile, as evidenced by the histogram‐, violin‐ and Q–Q‐plot shown in Figures S1 and S2, plotted from the same data as Figure 2c,d. Once the algorithm identifies groupings, it assigns labels to the clusters, and each data point receives the corresponding label. In Figure 2f, the result for an example of a bi‐particle mixture is shown: the cluster with lower LDV is labeled 0 and the one with higher LDV is labeled 1. Although our example contains only two clusters (a bi‐particle mixture), the number of clusters can be set to any natural number, allowing the analysis of mixtures with more than two particle types.

Now, the software has identified the clusters without any human intervention or prior training on similar datasets. The similarity in LDVs among particles within the same cluster suggests that they share similar surface chemistry. The next step is to identify the type of particles forming each cluster. This is done by comparing the cluster data points with those from single dispersions stored in the model library. While a human can easily infer that the faster particles are porous silica and the slower ones are non‐porous (Figure 2f), we aim to develop a software solution that performs this task automatically. Here we are in the same situation as before. We want to design a software solution for this problem, independent of a human. This becomes particularly important in more complex systems involving mixtures of multiple unknown particle types, where human analysis quickly reaches its limits.

**FIGURE 3:**
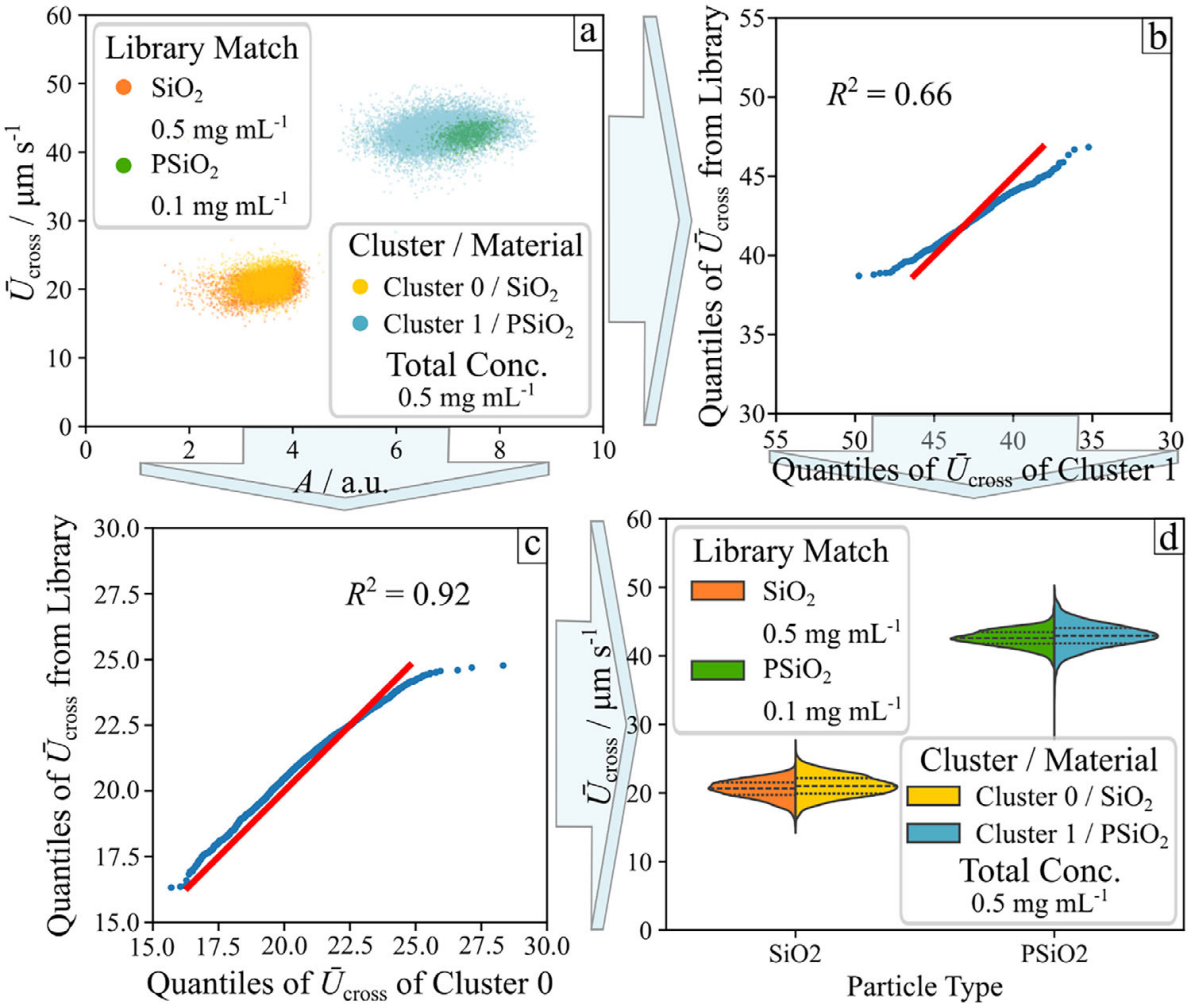
Example display of data identification by quantifying the best *R* ^2^ value from the quantiles of the velocity *U*_cross_ of library data against one cluster. A visualization of the matching algorithm comparing the data sets of the library (y‐axis) and analyte clusters (x‐axis) via Q–Q plots is shown in Video S3. (a) Clusters of measured data points library (same data as in Figure 2e). Data under light illumination. (b,c) Example fit of quantiles. (d) Calculated violin plot distributions of velocity *U*_cross_ classified by particles—library data and measurement. The dashed line illustrates the average value of *U*_cross_, the dotted line visualizes the 25%‐percentile and 75%‐percentile of the velocity distribution. The following sample sizes were determined: library data PSiO_2_ *n*_sample_ = 1217, library data SiO_2_ *n*_sample_ = 6734, analyte data *n*_sample_ = 20833.

To compare the clusters with the library, we use quantile–quantile (Q‐Q) plots of the *U*_cross_​ values. The library contains LDV measurements of single dispersions (SiO_2_ and PSiO_2_) at various particle concentrations (SiO_2_: 0.1–0.7 mg·mL^−1^, PSiO_2_: 0.05–0.7 mg·mL^−1^) with each concentration averaged over at least five repeated measurements. Details on the library and how to design the library in theory (categorizing) and in practice (data recording) are given in Section 4.3 and Section S12. Each cluster is compared to every dataset in the library by plotting Q–Q plots. Both datasets are divided into the same number of quantiles, where a quantile is a statistical value that divides a dataset into equal‐sized, ordered segments. Examples are shown in Figure 3c for plain silica (SiO_2_) and Figure 3b for porous silica (PSiO_2_). These examples represent the best matches from the quantile analysis between library and analyte clusters. Ideally, the Q‐Q plot of a library dataset versus an analyte cluster yields a linear relationship with a slope of one, known as the identity function  [33] (red line in Figure 3b,c). The best‐matching data points (blue) should closely follow this line. In practice, we compute Q–Q plots of the library relative to each cluster (Cluster 0/1), overlay the identity function (slope = 1), and compare the actual Q–Q data to this reference by calculating the *R* ^2^ value using Python (library numpy) [34]. This process is repeated in a loop for all clusters recorded under light illumination against all library datasets to identify the best match (i.e., the *R* ^2^ value closest to one) displayed in Video S3, which then informs the prediction of particle surface chemistry. To verify the quality of the Q–Q analysis, we also present a violin plot of *U*_cross_ for the identified clusters (0 and 1) alongside the pre‐classified library data for SiO_2_ and PSiO_2_ in Figure 3d. The similarity of average values and distributions confirms the potential of this method for automated particle detection.

It is crucial to emphasize that accurate quantification is only feasible when the particles are chemically active, that is, under light exposure. Illumination induces a sufficient velocity differential between the two particle populations, both in the reference library and within the binary mixture. In the absence of light, these velocity differentials are negligible and nearly overlapping, resulting in poor cluster separability. Consequently, clustering within the binary mixture becomes challenging, and matching with the reference library is unreliable.

This effect is illustrated in Figure S3 (Section S2). Despite the sample comprising a binary mixture of SiO_2_ and PSiO_2_, the classification algorithm misidentifies the particle types, assigning all particles to a single class SiO_2_. The regression‐based quantification yields suboptimal *R* ^2^ values of ‐0.82 and 0.52, indicating a poor model fit and highlighting the failure of classification under non‐illuminated conditions.

### 2.3 Quantification

In the previous section, we outlined the core methodology for the automated detection and classification of particle trajectories, enabling the interpretation of surface properties of both non‐porous and porous particles based on the measured LDV. The integration of real‐time velocity acquisition, computational data analysis, and subsequent interpretative modelling characterizes this approach. Within this context, we demonstrated a qualitative framework for analyzing individual particle trajectories as they were observed traversing the optical detection region of the microscope. In the current section, we describe the quantitative evaluation of the analyte by determining the particle number fractions *n*_clu,p_ of the two particle types from the number of detected particles and comparing them to theoretical particle number fractions *n*_theo,p_ calculated from the mass ratios known from sample preparation. The index *p* denotes the particle type: SiO_2_ for plain and PSiO_2_ for porous particles. Since each trajectory is already classified through the qualitative methodology, the number of trajectories detected is known for both particle types. They are determined by counting the trajectories labelled with the respective particle type. In the present case, these are the number of trajectories $N_{\mathrm{cross},\mathrm{Si}O_{2}}$ identified as SiO_2_ from Cluster 0 and $N_{\mathrm{cross},\mathrm{PSi}O_{2}}$ recognized as PSiO_2_ from Cluster 1. The counting is performed with the Python library *pandas*. The sum of both is the absolute number of microparticles crossing the detection area, *N*_cross_. Accordingly, for the two distinct clusters, representing plain and porous particles (Cluster 0 and Cluster 1, respectively, as shown in Figures 2 and 3), the fraction of each species can be computed using:

(3) $$n_{\mathrm{clu},\;p}=\frac{N_{\mathrm{cross},\;p}}{N_{\mathrm{cross}}}$$

**FIGURE 4:**
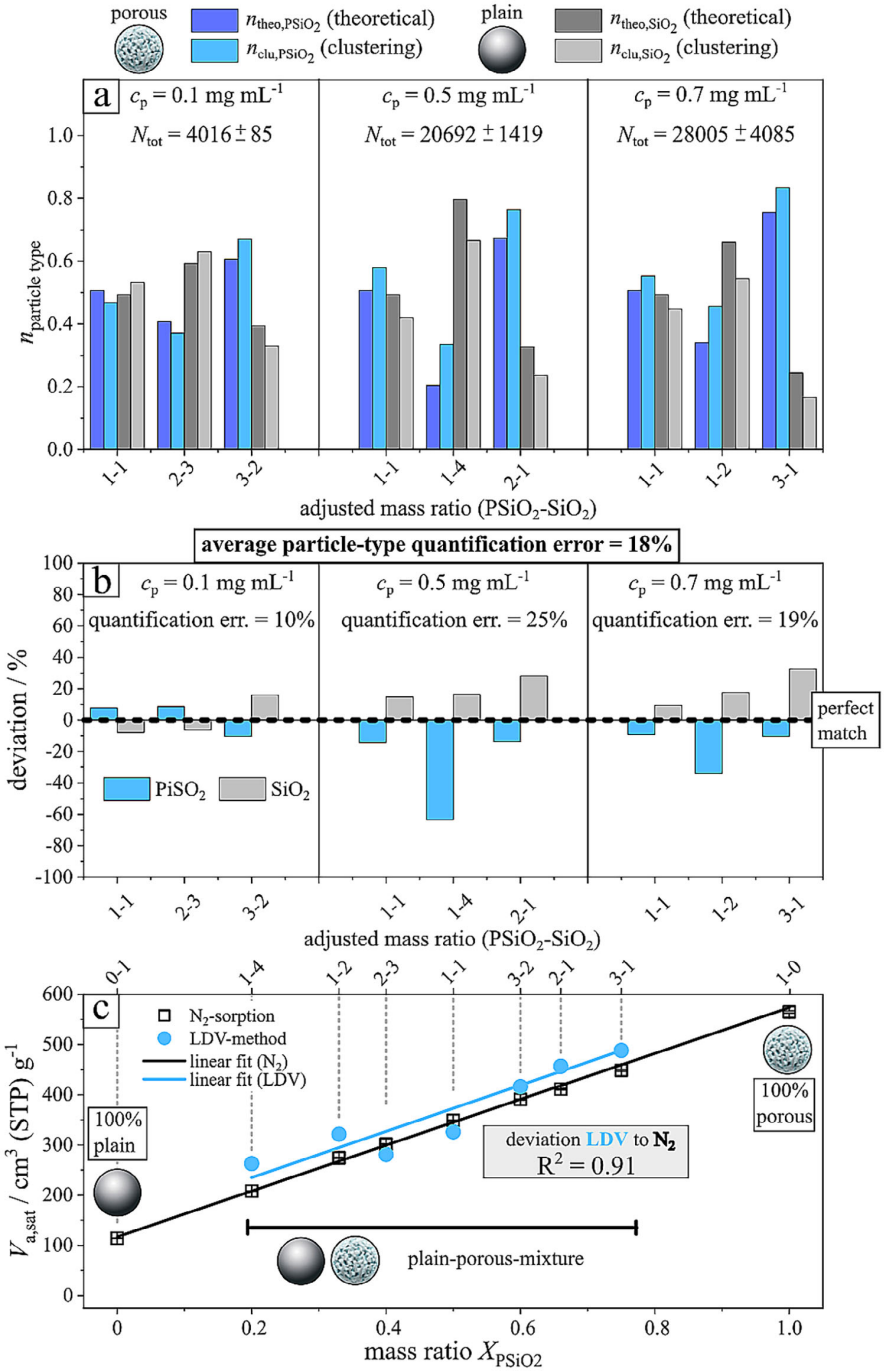
Quantitative assessment of measurement accuracy using method 1 (i). (a) Calculated number fraction *n* from the sample, plotted as a function of the theoretical mass‐based sample ratio and in total mass concentration. The total mass concentration and corresponding total number of particles are given in the figure. (b) Deviation of the calculated number fraction from the theoretical value, shown for the same sample classifications and total concentrations. (c) Measured and approximated nitrogen adsorption volumes at saturation (*V*_a,sat_) for the corresponding data sets. The average sample sizes classified by concentrations are 4016 ± 85 (*c*_P_ = 0.1 mg mL^−1^), 20692 ± 1419 (*c*_P_ = 0.5 mg mL^−1^), 28005 ± 4085 (*c*_P_ = 0.7 mg mL^−1^).

Here, the index p refers to the respective particle type SiO_2_ or PSiO_2_. We employed two principles of quantification, both yielding similar accuracy. The first principle (i) involves summing all trajectories classified by particle type. The second principle (ii) incorporates particle momentum by multiplying the number of particles detected per second by their average velocity ${\bar{U}}_{\mathrm{cross}}$. The key difference lies in the requirement of method 1 (i) to measure the entire sample (i.e., all particles must cross the detection area). In contrast, method 2 (ii) allows the total sample amount to be estimated based on momentum concentration, assuming a homogeneous distribution, thereby reducing measurement and analysis time. To further assess the accuracy and robustness of the quantification method, we systematically varied the mass ratios of non‐porous (plain) and porous particles within the analyte population. For these adjusted mass ratios, we computed the corresponding theoretical number fractions *n*_theo,p_ and then compared them to the experimentally determined fractions *n*_clu,p_ obtained from trajectory numbers $N_{\mathrm{cross},\mathrm{Si}O_{2}}$ and $N_{\mathrm{cross},\mathrm{PSi}O_{2}}$, as shown in Figure 4. We calculated the theoretical number fraction *n*_theo,p_ between both species from the adjusted mass ratio using the given material densities of the particles (∼1.8 g·cm^–^^3^ for PSiO_2_, ∼1.85 g·cm^–^^3^ for SiO_2_) and a particle diameter of 5 µm (see calculations in the Supporting Information). Since the imaging method yields particle number fractions, we only computed these fractions, as displayed in Figure 4a. Additionally, we measured three total mass concentrations *c*_total_, 0.1, 0.5, and 0.7 mg·mL^−1^. For each concentration, we randomly selected three different adjusted mass ratios of PSiO_2_ and SiO_2_. Thus, both the total mass concentration and the mass ratio were systematically varied. To evaluate the accuracy of total particle concentration, we computed the absolute number of microparticles crossing the detection area *N*_cross_ by summing $N_{\mathrm{cross},\mathrm{Si}O_{2}}$ and $N_{\mathrm{cross},\mathrm{PSi}O_{2}}$. The data revealed a strong correlation between adjusted and measured total particle concentrations. Over a 35‐second recording period, the following trajectory counts were detected: 4016 ± 85 at *c*_total_ = 0.1 mg·mL^−1^, 20692 ± 1419 at 0.5 mg·mL^−1^, and 28005 ± 4085 at 0.7 mg·mL^−1^. Upon normalization to the lowest concentration (0.1 mg·mL^−1^), we obtained the following relative concentrations: 1.00, 5.15, and 6.92, which are in good agreement with the adjusted particle concentration ratio 1:5:7. Furthermore, to assess the accuracy of particle number fraction determination in a binary mixture, we analyzed the trajectories classified into non‐porous and porous particles. The accuracy was determined by first calculating the deviation between *n*_theo,p_ and *n*_clu,p_, and the average deviation, as described in the Supporting Information Section S6. From the average deviation over all analyte measurements, we calculated an average particle‐type quantification error η_pq_ of 80%, and a low average particle‐type quantification error of around 20% was achieved (for calculation see SI) for both quantification methods: method 1 (i) (Figure 4b) and method 2 (ii) (Figure S7b). The measured values of the particle number fraction *n*_clu,p_ for porous (PSiO_2_) and plain (SiO_2_) particles remained consistently close to the expected theoretical number fraction *n*_theo,p_ values across all concentrations (see Figure 4a). This is evidenced by the low average particle‐type quantification error close to 20% for methods 1 and 2. Overall, the data presented in Figure 4a,b demonstrates the potential of this approach for quantifying total particle concentration via trajectory counts, as well as determining particle number fractions in binary mixtures through trajectory identification using the developed algorithm. To conclude, the two principles of quantification are revisited. In the first method (i), all tracks are counted, and a direct measurement of the sample is provided, trading off a smaller scanning area of the objective against the width of the microfluidic channel. In the second method (ii), the momentum flux of incoming and outgoing particles is approximated, and the total mass is estimated by multiplying the flux by the total measurement time. Quantification results shown in Figure 4 and Figure S7 indicate that both approaches yield reliable outcomes, for the fraction calculated from the number of classified trajectories (*n*_clu,p_) compared to the theoretical number fraction (*n*_theo,p_) obtained by the adjusted ratios (with particle‐type quantification errors of approximately 20%).

The proposed method enables the quantification of the average porosity of the macroscopic sample by evaluating a weighted distribution of particle surface areas derived from measured particle trajectories. Specifically, this is achieved through a linear combination of the parameters ***X***, which denotes the characteristic surface area associated with either fully porous or fully non‐porous microparticles. The weighting factors correspond to the relative frequency or proportion of each particle type within the measured population, thereby providing an estimate of the overall porosity based on the compositional heterogeneity of the sample given by:

(4) $$X_{\mathrm{average}}=n_{0}\cdot X_{\mathrm{Si}O_{2}}+n_{1}\cdot X_{\mathrm{PSi}O_{2}}$$

Here, *X* represents the apparent measured quantity associated with surface area, such as the adsorbed nitrogen volume *V*_a_, the specific surface area determined via the BET method, or any other surface‐sensitive parameter classified according to Clusters 0 and 1. These values are further weighted by the experimentally recorded fractions *n* of particle trajectories from crossing velocities associated with each respective cluster. It is important to note that indices 0 and 1 refer to Cluster 0 (non‐porous) and Cluster 1 (porous), respectively.

To validate the accuracy of this quantification approach, we conducted experimental comparisons using nitrogen adsorption measurements on the same dried binary mixtures of porous and non‐porous particles (with total mass ∼100 mg). The results, as illustrated in Figure 4c, demonstrate strong experimental agreement between determined porosity values. The adsorbed saturation volume *V*_a,sat_ for the pure porous particle sample, as shown in Figure S8, reveals a multiporous silica matrix structure. This is evidenced by a dual‐stage saturation behavior at distinct relative pressures, with a maximum adsorption of 565 cm^3^·g^−1^ observed in the normalized pressure range Δ*p*/*p*_0_ = 0.8–1.0, indicative of the presence of macropores. In contrast, the non‐porous particle sample exhibits a single‐stage (monophasic) saturation curve, reflecting a smoother surface morphology and the absence of larger pore structures. Nevertheless, a measurable *V*_a,sat_ value of approximately 114 cm^3^·g^−1^ is recorded, which is attributed to the intrinsic mesoporosity of silica; even non‐porous silica particles can permit limited nitrogen adsorption due to the penetration of nitrogen molecules into residual mesoporous regions. For binary particle mixtures, the measured *V*_a,sat_ at **Δ** ***p***/***p***_0_ = 0.8–1.0 increases linearly with the proportion of porous particles in the sample (see Figure 4c, hollow dark data points; raw data available in Figure S9). This linear relationship confirms that the macroscopic porosity of the composite sample scales directly with the population of porous particles. To compare the LDV method with conventional nitrogen sorption isotherm analysis, we employ Equation (4), incorporating the number fractions $n_{0}=n_{\mathrm{Si}O_{2}}$ and $n_{1}=n_{\mathrm{PSi}O_{2}}$, as obtained from the trajectory classification shown in Figure 4b. The parameter ***X*** is assigned to the adsorbed saturation volume *V*_a,sat_, with values of 114 cm^3^·g^−1^ for pure SiO_2_ (non‐porous) and 564 cm^3^·g^−1^ for pure porous PSiO_2_. Using these values, the weighted average *V*_a,sat_ was computed for the various binary particle mixtures depicted in Figure 4a,b, and the results are presented in Figure 4c (blue data points). The LDV‐based predictions show excellent agreement with experimentally obtained nitrogen sorption data, with an average absolute deviation of only 9%. A similar approach was applied to the second independent quantification method (ii) (see Figure S7 and Section S6), which resulted in a slightly higher deviation of 16%. These results confirm that the population composition of the particle mixtures can be reliably determined from the LDV‐measured velocity trajectories, thereby supporting the robustness and accuracy of the LDV method for quantifying macroscopic sample properties.

#### 2.3.1 Surface Area Approximation via Population Knowledge

Analogously, the average specific surface area of the sample can be approximated, provided that the surface areas of the reference particles are known. For instance, the surface area of the porous particles was measured from the company supplier to be 850 m^2^·g^−1^ (BET value), whereas the non‐porous SiO_2_ particles, assumed to be perfectly smooth and spherical, yield an estimated surface area of 0.54 m^2^·g^−1^. By applying Equation (4) with the same number fractions, a progressive increase in the calculated average surface area is observed across the mixture series, as summarized in Table 1.

To conclude, we revisit the two principles of quantification. The first method counts all tracks and provides a direct measurement of the sample, trading off a smaller scanning area of the objective against the width of the microfluidic channel. The second method approximates the momentum flux of incoming and outgoing particles and estimates the total mass by multiplying the flux by the total measurement time. Quantification results shown in Figure 4 and Figure S7 demonstrate that both approaches yield reliable results, either for adjusted versus measured number ratios (with errors around 20%) or for computed versus measured N_2_‐sorption (with deviations below 9%).

### 2.4 Measurement Advantages

The LDV method offers several practical and analytical advantages over conventional nitrogen sorption techniques for characterizing porous and non‐porous particle mixtures, some of which are listed and explained in more detailly below:

1) *Minimal Sample Quantity*: The LDV technique requires significantly smaller sample mass (sub‐milligram to milligram scale) compared to the ∼100 mg typically needed for reliable nitrogen sorption. This is particularly beneficial for rare, expensive, or difficult‐to‐synthesize materials. Since surface area is indirectly measured via particle velocity, the quantity of observable particles themselves defines the minimum required sample amount. For example, the mass of the measured sample is calculated as the number of particles multiplied by their volume (*V* = 4/3 · π · *a* ^3^) and their density (*ρ *∼ 1.85 g·cm^−3^). For the lowest concentration, the analyzed sample mass is approximately 4.9 · 10^−7^ g (or 4.9· 10^−4^ mg). The theoretical minimum sensitivity is defined by the requirement that at least one particle must be detected. Accordingly, the sample mass can be as low as the mass of a single particle, which is 1.2 · 10^−10^ g (or 1.2 · 10^−7^ mg). In contrast, a typical nitrogen sorption measurement requires at least 100–200 mg of the sample, depending on porosity, representing a difference of nine orders of magnitude. This highlights the LDV method's suitability for small‐batch analysis.
2) *Rapid Data Acquisition*: LDV provides fast characterization, with velocity trajectories of individual particles being recorded and classified within seconds to minutes. Using the first quantification approach, based on counting the full population of particle trajectories, reliable results can be obtained with a minimum recording duration of approximately 45 s. However, when employing the second quantification approach, which considers only the net in‐ and outflux of particles within the image window, the acquisition time can be reduced to 5 seconds. This comes at the cost of slightly reduced accuracy, as reflected in the higher average deviation (16% vs. 9%) from nitrogen sorption data. The 5 s threshold is established based on the average transit time required for a single particle to cross the optical field of view under typical flow conditions. This duration can be further shortened by increasing the applied shear rate, which accelerates particle velocity through the measurement zone. When directly comparing measurement times, nitrogen sorption requires between 8 and 43 h per sample, depending on material porosity and the resolution of the isotherm. In contrast, the LDV method yields comparable quantitative results within a timeframe on the order of seconds, representing a reduction of approximately four orders of magnitude in time. This dramatic improvement in temporal efficiency highlights the strong potential of the LDV method for integration into real‐time or online analytical systems, particularly for process monitoring and rapid quality control in industrial or laboratory environments.
3) *Particle‐Based Resolution*: The LDV method captures individual particle behavior, allowing direct assessment of heterogeneity within a sample. Nitrogen sorption, by contrast, provides only bulk‐averaged properties, limiting its capacity to resolve bimodal or polydisperse systems without extensive deconvolution.
4) *Versatile Application*: The LDV approach is broadly applicable to a wide range of particle types and suspensions. We emphasize that the examples presented here were based on 5 µm silica particles. However, the physical phenomenon of the *l*‐LDDO applies to any interface [19]. This includes porous surfaces, as well as variations in surface modifications for equally sized particles such as surface functional groups [24, 26], polymer brush coatings [35], and microgels ranging in size from 2 to 40 µm in diameter [36, 37]. Thus, we expect the detection principle to be broadly applicable, as it is based on recording light‐induced drift motions. However, for different particle types, the reference library must be calibrated accordingly.
5) *Porous Colloids—Porosity Determination of Internal or External Surface Area*: As surfactants primarily interact at interfaces and with the surrounding bulk solution, the external surface area generally represents the dominant contribution. However, if the external surface allows surfactant diffusion into the internal structure via sufficiently large pore diameters, the internal surface area may also become relevant. Given that the characteristic length of the surfactant molecules is approximately 2 nm [38], we expect adequate pore diffusion and correspondingly rapid isomer exchange for pore diameters of at least 4 nm or larger, thereby maintaining chemical activity proportional to the accessible surface area. It should be noted that this behavior is highly sensitive to the ratio of surfactant dimensions to pore size and thus the strength of chemical activity and corresponding detection may rely on apparent pore sizes of dispersed microparticles.

It is important to emphasize that the data science approach employed in this study represents only one of several possibilities. Specifically, we used unsupervised machine learning techniques, which are well‐suited to our dataset of unlabeled particle trajectories. Alternative data science methodologies potentially offer improved performance in terms of classification accuracy, quantitative analysis, and computational efficiency, which probably exist and may be explored in future work. For example, several AI‐based detection methods have already been demonstrated for a range of modern optical sorting mechanisms, as described elsewhere. In future work, selected AI algorithms could be further integrated into the data processing pipeline to enhance object detection and outlier removal [39]. Additionally, such approaches may be applied at the data analysis and interpretation stage, for example, by enabling trajectory and velocity comparisons in support of chemical identification. Nonetheless, the core concept has been successfully demonstrated: particle trajectories acquired under controlled illumination and fluid flow can be compared to a reference library of pre‐recorded trajectories obtained under identical system configurations to predict the surface properties of individual microparticles. Due to its low hardware demands, this method offers a robust and cost‐efficient solution for particle‐based characterization of microparticles suitable for determining porosity and surface area per particle and as the entire sample amount for small‐batch analysis.

## 3 Conclusions

This work introduces a novel analytical technique for particle‐based porosity determination using optical video microscopy. The measurement principle is based on recording the size and passive motion of sedimented particles in an external flow. The dispersion contains a light‐responsive surfactant that renders all colloids active upon illumination. Under an external flow, this activity results in unique particle velocities in the direction of the streamlines, which are characteristic of the particles’ chemical and interfacial properties. The recorded trajectories can be analyzed and compared with a predefined data library to determine the chemical and interfacial properties of each crossing particle. Since this is an imaging‐based method, it simultaneously measures both the physical properties (e.g., size and size distribution) and the chemical/interfacial properties of the particles; that is, it follows the principle of particle‐based detection.

More specifically, the analysis follows a four‐step process: (I) simple, consistent sample preparation, (II) data acquisition using optical video microscopy combined with microfluidics and LED illumination, (III) trajectory analysis using a data processing pipeline, and (IV) final reporting for qualitative and quantitative evaluation of particle type and total concentration.

In step (I), particles are dispersed in a photosensitive azobenzene‐containing surfactant. Then, in step (II), the mixed dispersion is injected into a microfluidic chamber and measured under flow while being illuminated. Step (III) involves object detection and particle tracking using standard tracking algorithms to compute particle velocities from their trajectories. After outlier removal, the dataset reveals distinct accumulations of data points, where particles with similar sizes and velocities form localized clusters. These natural groupings can be identified using standard unsupervised machine learning algorithms, such as the Gaussian Mixture Model (GMM). The resulting clusters are compared with clusters of a pre‐defined data library of known particle composition via the velocity values. This comparative analysis enables the prediction of the interfacial properties of analyte particles and forms part of the reporting framework (Section IV). Furthermore, when summed across the entire sample, the qualitatively classified and interpreted individual trajectories allow for the determination of the overall sample composition in terms of particle types and their distribution within the population. The analytical principle is demonstrated using model silica microparticles with plain and porous interfaces. We evaluated the accuracy of particle‐based analysis by comparing adjusted and measured sample compositions and found an average accuracy to 20%. Additionally, we estimated the effective net surface area of the bi‐particle mixture by calculating a weighted average of the BET values of plain and porous particles, based on their population ratios. This estimate closely matched the net surface area obtained from conventional BET analysis under identical sample compositions.

## 4 Careful Points to Consider

### 4.1 Sample Preparation

Ensuring precise and consistent mixing of the particles with the surfactant solution is essential for both library and analyte measurements, as particle phoretic mobility and light‐induced drift are highly sensitive to surfactant concentration, ionic strength, and sample preparation. Small deviations in absolute velocity can result in significant discrepancies when comparing data clusters. For sample preparation, microparticles are first washed with Millipore water and then suspended in an aqueous solution of the azobenzene‐based surfactant at the desired final concentration. A detailed schematic of the washing cycle is provided in Figure S16 (Supporting Information), illustrating the procedure.

I. Disperse the particles in the solvent (water or aqueous surfactant solution) by sonicating them for 1 min.
II. Allow the particles to equilibrate in the solvent for at least 5 min.
III. Remove/replace the supernatant by first centrifugation with subsequent removal of the supernatant.
IV. Immediately re‐disperse the particles, ensuring they remain wet throughout the process.
V. After transferring the particles to the solution of the photoswitchable surfactant AzoC_6_, store them for further equilibration (e.g., 1 day), avoiding exposure to light or heat during this period until the final analysis.

**FIGURE 5:**
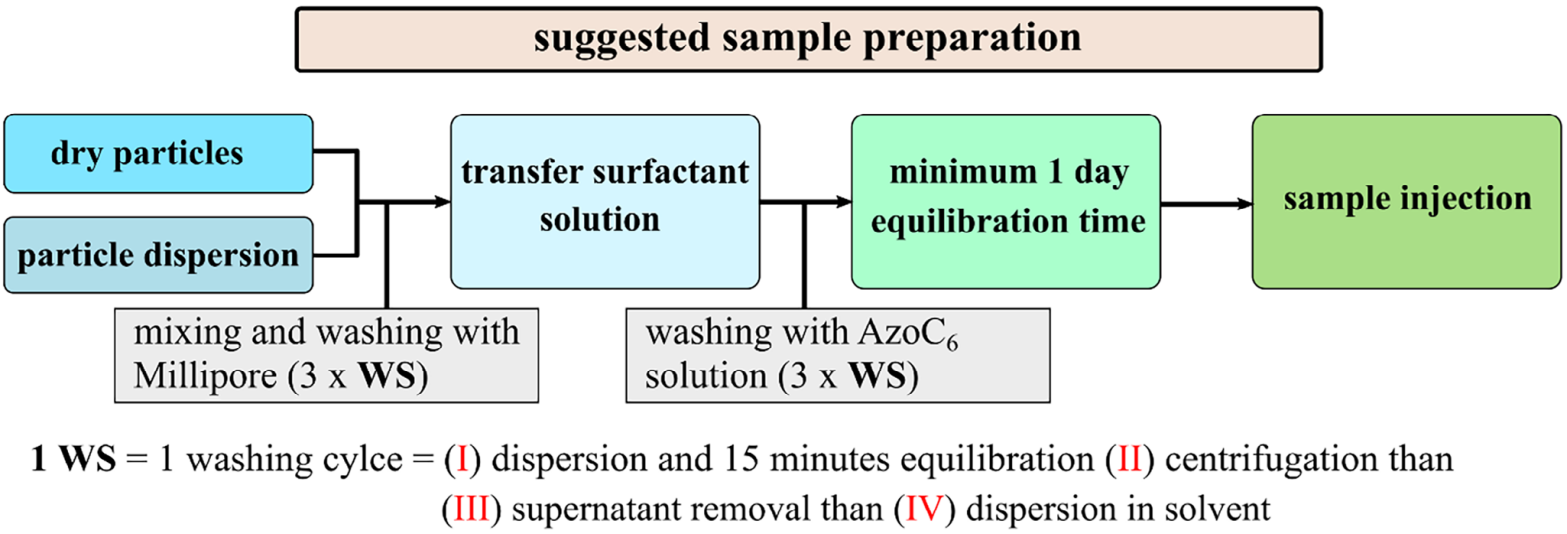
Detailed preparation protocols for making the samples. It is important to maintain consistency, due to the light‐induced activity, the *local*‐LDDO [24, 40], is very sensitive to the magnitude of photosenstive surfactant exchange at interfaces. It is important to have the same preparation protocol for data library design and sample measurement.

The examples described above, along with the schematic in Figure 5 and the detailed protocol in Figure S16 and Section S11, serve as guidelines. The number of washing cycles and equilibration times may be adjusted, but must remain consistent across all measurements, for both the library and analyte. Inconsistencies in preparation can result in mismatched light‐induced velocities and lead to misidentification. As a rule of thumb, we recommend three washing cycles with Millipore water and the photosensitive surfactant AzoC_6_ solution (see Figure 5).

### 4.2 Outlier Removal

**FIGURE 6:**
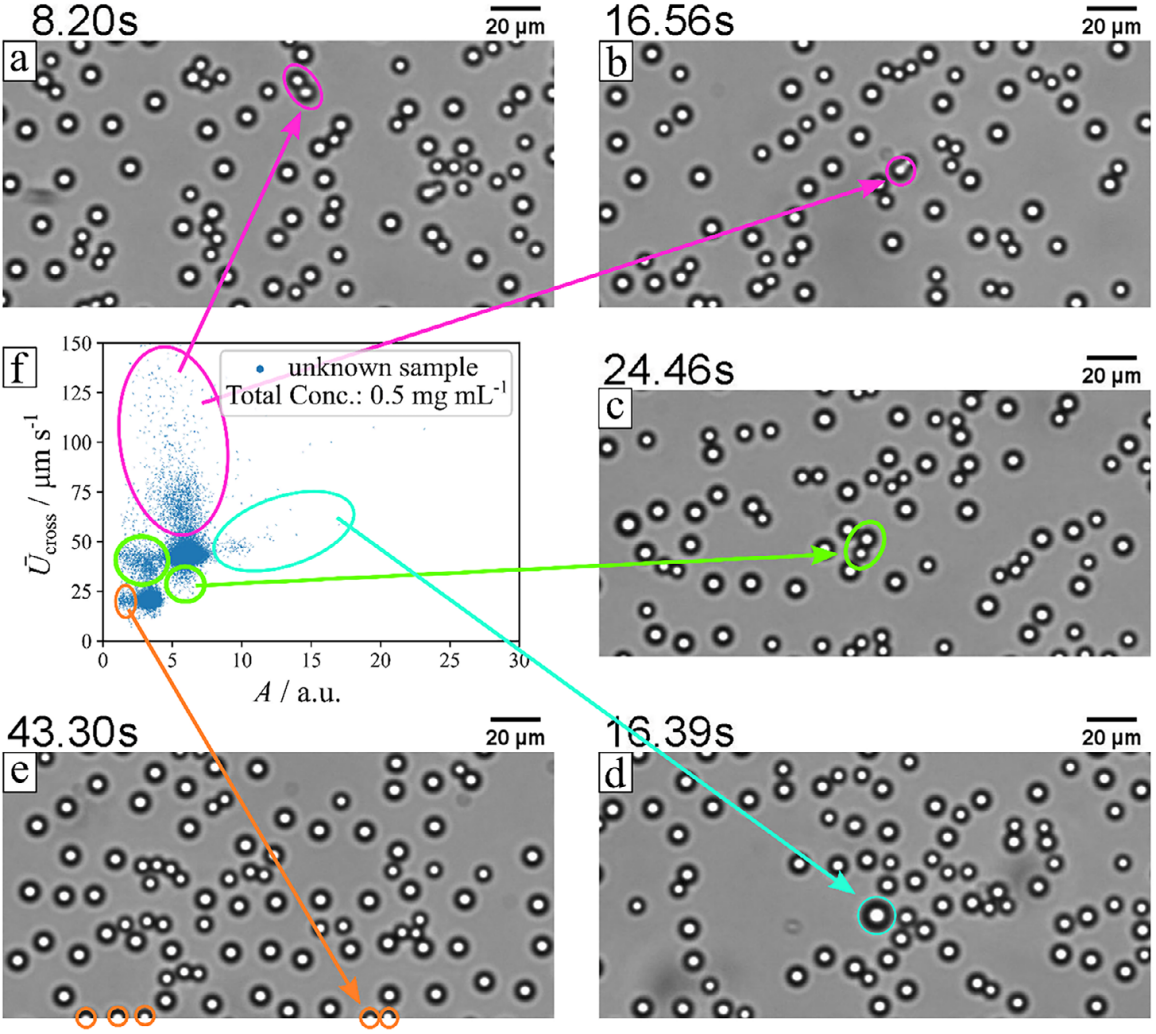
Visual examples of outlier removal. Example data from Video S4 (a,c) aggregated particle doublets or multiples, (b) asymmetric deformed particles. (d) Big‐sized particles. (e) Too small, the misidentified size of particles recorded at the edge of the image. (f) *U*_cross_ as a function of the recorded particle sizes in pixel area. Data display without outlier evaluation (*n*_sample_ = 43014). The color code of ellipsoids and arrows corresponds to labelling in the images.

As described in Section 2.3, outliers must be removed prior to clustering, as they result from mistracking, sample impurities, or particle clusters, and can generate additional clusters that compromise clustering performance. Four types of outliers were identified (Figure 6, Video S4): colliding particles, particles with abnormally large diameters, particles entering or leaving the field of view, and particle clusters. Collision‐induced mistracking produces fragmented, short tracks with anomalously high velocities, which were removed using a path length ≤0.15 quantile and a velocity standard deviation ≥0.9 quantile. Clusters containing particles of different materials create outliers due to differential phoretic activity; these were removed manually, with future AI‐based cluster analysis suggested for automation. Edge particles appear smaller or produce fragmented tracks and were treated like collision outliers. Finally, particles with areas above the 0.99 quantile were removed to exclude unusually large, fast‐moving particles. More details are shown in Supporting Information (Section S12.2).

### 4.3 Library Design

The core of the particle identification relies on the comparison between the quantiles of *U*_cross_ from the library and the analyte data. To maximize identification accuracy from quantile comparison, it is essential to achieve a close match between crossing velocities *U*_cross_ of both datasets.

In general, the library must be constructed using the same measurement conditions as the analyte, with the key difference being that the physical (e.g., size, shape) and chemical (e.g., material, surface functionalization, porosity) properties of the particles in the library are well known and clearly classified. Data acquisition for the library involves recording trajectories of the particles following the flow streamlines under light illumination at fixed wavelength and intensity, ideally for one particle species at a time, and importantly, across a range of particle concentrations. This variation is crucial for covering the expected concentration range of the analyte in bi‐particle mixtures. To better understand the design of the library and the core parameters used for classification, we briefly explain the physical phenomenon behind the LDV dependence (∼*U*_cross_ change as recording parameter) on parameters such as flow rate, particle size, particle surface chemistry [24, 25, 35], intensity [41], wavelength [26] and recently reported also on the particle concentration [32].

#### 4.3.1 Flow Rate, Intensity, Wavelength

Light intensity and wavelength influence the dynamic exchange of the photo‐sensitive surfactant isomer [19], which in turn affects the strength of particle activity [26]. This activity determines the lift‐off behavior, while the shear rate *S* (dependent on flow rate and cell geometry) modulates the magnitude of LDV for all particles equally. In practice, flow rate, intensity, and wavelength can be precisely controlled and are kept constant across both library and analyte measurements.  As a result, only a negligible variation in *U* is expected between recordings.

#### 4.3.2 Particle Size

Particle size has a strong influence on the magnitude of *U*, larger particles generally exhibit higher velocities. This applies to both fully sedimented and lifted particles under light illumination [41]. Thus, it is important to classify *U*_cross_ in the library according to particle size at a given surface chemistry.  For demonstration purposes, we used a simple system with particles of uniform size (5 µm diameter) and varied only their surface area by comparing plain and porous particles.

#### 4.3.3 Particle Surface Chemistry

The strength of light‐induced activity is highly sensitive to interfacial properties, including surface functionalization, bulk material, surface area, and surface charge. These factors influence the surfactant's storage capacity and exchange dynamics upon illumination. For further details, we refer the reader to relevant literature [19, 24, 26, 32, 35, 41]. Consequently,  it is critical to classify the library by interfacial properties such as surface area (e.g., plain vs. porous), surface functional groups (e.g., –OH, –NH_2_, –NR_3_^+^, –C_18_), and particle material (e.g., silica, polystyrene, polymethacrylic acid).

In our demonstration, we intentionally fixed most surface chemistry parameters. Both particles shared the same particle bulk material (silica) and surface functionalization (–OH). The only variation was the effective surface area, allowing us to classify particles based solely on porosity type–i.e., plain versus porous surface.

#### 4.3.4 Particle Concentration

We recently demonstrated that the strength of particle activity depends on the distance to the nearest neighboring active particle. This is because adjacent particles generate spatial *cis*‐isomer gradients, which, when overlapping, reduce the net activity of each particle. As particle concentration increases, the average interparticle distance decreases, leading to stronger gradient overlap and, consequently, a reduction in activity. This results in a lower levitation tendency and a corresponding decrease in *U*_cross_ [32].

In practice, natural analyte samples rarely exhibit uniform particle concentrations, neither in absolute terms nor in the relative proportions of different particle types. This is important because the activity of one colloid is influenced by the neighboring colloids [32]. Assuming a bi‐particle mixture contains both strongly and weakly active colloids, their mutual repulsion via *l*‐LDDO depends on their relative positions and activity strengths. Weak–weak pairs can approach closely before repulsion sets in, while strong–strong pairs maintain greater distances. Mixed strong–weak pairs align somewhere in between. While simplified, this model illustrates the complex collective behavior: the closer the particles (i.e., the higher the concentration), the more their activity is influenced by neighboring particles. The effective strength of *l*‐LDDO and the resulting LDV both decrease with increasing particle concentration under fixed illumination [32].

This concentration‐dependent behavior causes deviations in LDV between single dispersions recorded in the library and bi‐particle mixtures in the analyte, even at the same total particle concentration. To minimize such discrepancies, the library must be designed to match the individual particle concentrations of the analyte, not just the total concentration. We propose a simple technical solution to address this issue.

If we assume that only weak‐weak or strong‐strong interactions dominate, active particles are interacting, neglecting interactions between weak and strong active particles, then the relevant factor for LDV (∼*U*_cross_​) is the absolute concentration of each particle species. For an analyte composed of two finite particle types, labelled Cluster 0 and 1, the total particle concentration *c*_tot,analyte_ is:

(5) $$c_{\mathrm{tot},\;\mathrm{analyte}}=c_{0,\;\mathrm{analyte}}+c_{1,\;\mathrm{analyte}}$$

where *c*_0_ and *c*_1_ are the concentrations of each particle type is a simple sum from ensembles of Cluster 0 and 1 with corresponding particle concentration *c*_0_ and *c*_1_, where the indices 0 and 1 represent the particle type in the analyte. For the library, where *U*_cross_ from the particle mixture only a single dispersion of particles with well‐known surface properties is measured, the total concentration *c*_tot,library_ is:

(6) $$c_{\mathrm{tot},\;\mathrm{library}}=c_{X,\;\mathrm{library}}$$

with *X* = 0 or 1 representing the particle type. The LDV values (∼*U*_cross_) are only comparable if the condition *c*_0,library _∼ *c*_0,analyte_ and *c*_1,library _∼ *c*_1,analyte_ is satisfied. Since the ratio of Cluster 0 to Cluster 1 may vary between analyte samples, the corresponding concentrations *c*_0,analyte,_ and *c*_1,analyte_ must also vary. Therefore, it is essential to compare the LDV values of the clusters with datasets from the library, whose total concentration is similar to the absolute concentration of the species in the mixture, *c*_x,library _∼ *c*_ x,analyte_, rather than compare library and analyte by the total concentration. At equal total concentrations, particles in a mixture may exhibit faster LDV than in the library because the concentration of each species is lower, that is, more diluted, resulting in fewer particle–particle interactions and stronger individual activity. This leads to stronger levitation and faster LDV in the analyte compared to the library [32]. Experimental data in Figure S10 and Section S8  (Supporting Information) support this, showing that *U*_cross_ values from the analyte only match those from the library when the library data is taken at a lower particle concentration.

Since analyte samples may vary in both total concentration and particle composition, iterating the library across a range of particle concentrations is critical for accurate *U*_cross_ matching. This is one of the most important considerations in library design. Principles are illustrated in Figure S10. By considering that we measured the value of *U*_cross_ for every single dispersion, i.e., plain and porous silica colloids in a concentration from *c*_p_ = 0.1–0.7 mg·mL^−1^, where the data is plotted for both particles in Figure S11 and S12. Every library dataset at one fixed concentration contains 5 individual measurements of 30 s recording time. Data in Figure S11 exhibits that the last 3 out of 5 data sets show a constant *U*_cross_. These are combined and represent the library data set at one particle concentration. The same iterations are done with all other concentrations.

### 4.4 Long‐Term Stability of the Library

The crucial prerequisite for the long‐term stability of the library is the reproducibility of the measurements. It must be ensured that the once‐measured library data captures the properties of the particles represented. This can be split into two requirements: the reproducibility of the experiment with respect to physical and chemical conditions, and the stability and adjustment of the measurement equipment, ensuring consistent observation parameters.

This first point requires consistent sample preparation. We suggest the one that is described in detail in Section 4.1. Moreover, to ensure comparable strength of chemical activity of the particles, it is mandatory that the photosensitive surfactant concentration remains constant, apparently recommending a concentration of 2 mM, which provides the best stability with respect to chemical activity. In addition to chemical parameters, the same physical parameters must be established. This means that the fluid dynamic properties of the microfluidic setup must remain consistent for all library and analyte measurements. This includes the pump type, ensuring the stability of the flow rate as discussed in Section 4.5, and a constant flow rate for all measurements.

The second point is crucial for the transferability of the library. To ensure a reliable match between the library data and the data of a measured analyte sample. It is crucial that the setup used to measure the analyte is an exact clone of the one used to measure the library in terms of optical properties, excitation wavelength, intensity and microfluidics. This is necessary because determining particle size and velocity via tracking requires a consistent set of optical properties, ensuring a fixed focal plane position and a constant depth of field. This guarantees that the particle area measured in both cases is comparable, and all particles are tracked without losing track due to a small depth of field.

### 4.5 Pump Type

A critical parameter for reliable material identification using LDV is the pumping system and its flow rate stability. Accurate and repeatable delivery of a constant flow, particularly over millisecond timescales, ensures consistent hydrodynamic conditions. In this work, a high‐precision syringe pump was used, providing low, steady flow rates with minimal pulsation and noise, maintaining a uniform pressure gradient essential for consistent particle motion. In contrast, peristaltic or gear pumps inherently produce pulsatile flows, causing transient accelerations and decelerations that lead to non‐uniform particle motion, broad overlapping data clusters, and potential misclassification. Video S5 and Figure S13 compare a syringe pump with two peristaltic pumps, showing that while average flow rates converge, instantaneous velocity profiles differ markedly. Pulsations in peristaltic pumps generate motion blur, longitudinal smearing, and apparent particle size variations, resulting in erroneous outliers. The syringe pump, by contrast, produces compact, well‐separated data clusters, ensuring reliable material classification. For optimal performance, high‐precision pumps with minimal pulsation should be used, and all fluid components must be uniformly filled to prevent dilution in the microfluidic chamber. More details are shown in Supporting Information (Section S12.4).

### 4.6 Image Acquisition Time

Accurate data interpretation depends critically on video acquisition, as long exposure times can cause fast‐moving particles to appear smeared, leading to mis‐measured sizes and misclassified data clusters. To minimize motion blur and improve temporal resolution, exposure times should be as short as possible. We compared two exposure settings (33 ms and 1 ms) at a constant frame rate of 30 FPS, keeping all other acquisition parameters constant. For the short exposure, red LED illumination (*λ* = 625 nm) intensity was increased to maintain consistent image brightness.

A demonstration is shown in Video S6, where porous microparticles are illuminated with UV light (*λ* = 365 nm) to provide the maximum boost velocity [42] from the fastest photoisomerization kinetics at a given wavelength [40, 43, 44]. Figure S14 and Video S6 show snapshots under dark and light conditions, comparing real images and thresholded binary representations at different exposure times. Prolonged exposures cause motion blur, producing elongated particle shapes along the motion direction, especially under illumination. Short exposures preserve the true spherical geometry. Importantly, measured particle velocities remain consistent across exposure times (Figure S15), as particles traverse the same distance between frames (30 FPS). However, long exposures artificially increase calculated pixel areas and apparent aspect ratios, introducing size artifacts. Motion‐induced broadening or shifting of data clusters in *U*_cross_ versus particle area can lead to misclassification, introducing bias by reflecting apparent rather than true particle size. Accurate size determination, independent of particle velocity, is achieved using a short exposure of 1 ms, while maintaining a 33 ms inter‐frame interval (30 FPS). This minimizes motion blur, enabling reliable morphological analysis of both slow‐ and fast‐moving particles, and reduces frame loss or temporal artifacts caused by pixel readout delays. The red LED illumination (*λ* = 625 nm) does not induce photo‐isomerization, allowing higher intensity to compensate for the short exposure and maintain optimal image brightness and signal‐to‐noise ratio. For more information, see Section S12.5.

## 5 Experimental Section/Methods

### 5.1 Materials

*Monodisperse mesoporous silica colloids* (referred as “porous” silica) with an average particle diameter of *D* = (5.0 ± 1.0) µm and non‐porous, solid silica colloids (“plain” silica) with an average diameter of *D* = (5.0 ± 0.24) µm, were obtained from Micromod Partikeltechnologie GmbH (Rostock, Germany) and microParticles GmbH (Germany), respectively. Both types of colloids were supplied as aqueous suspensions without additional surface functionalization. The porous silica colloids exhibit a well‐defined mesostructured, characterised by a high internal surface area (company measure surface area BET = 850 m^2^·g^−1^) and accessible pore networks for the photosensitive surfactant, whereas the plain silica particles serve as a non‐porous counterpart.

#### 5.1.1 Azobenzene‐Containing Surfactant

We synthesized the azobenzene‐containing trimethylammonium bromide surfactant (C_4_‐Azo‐OC_6_TMAB) according to the protocol as described elsewhere [45]. The surfactant (Figure 1b) consists of a spacer of 6 methylene groups between the positively charged trimethylammonium bromide head group and the azobenzene unit with a butyl tail attached [44]. An aqueous surfactant stock solution with a concentration of 10 mM was prepared that was diluted to 2 mM during sample preparation, together with the addition of microparticles.

### 5.2 Experimental Methods

#### 5.2.1 Sample Preparation

Aqueous dispersions of non‐porous (SiO_2_) and porous silica (PSiO_2_; pore diameter: 12 nm) microparticles with a diameter of *D* = 5 µm (see Figure 1c,d) were mixed with a 2 mM surfactant solution, far above the critical micelle concentration (cmc_surfactant_ = 0.5 mM) [46]. Milli‐Q water with a specific resistance greater than 18 MΩ·cm was used to prepare aqueous solutions. Binary mixtures of porous and non‐porous silica microparticle solutions were prepared at different concentrations (0.1, 0.5, and 0.7 mg·mL^−1^) and ratios (1:1; 2:1; 1:2; 3:2; 2:3; 1:4). The mixture was allowed to equilibrate for at least 24 h prior to performing flow measurements. To prevent unwanted photo‐isomerization, we kept all samples in the dark or under red light. We injected the aqueous dispersion into the microfluidic chamber, where the colloids sedimented onto the glass surface. A µ‐Slide^VI^ glass chamber (Ibidi GmbH, Gräfelfing, Germany, *n*_D_ = 1.523) was used to provide the closed environment. The chamber is connected to a syringe pump (PhD Ultra, Harvard Apparatus). The volumetric flow rate was fixed to 150 µL·min^−1^ and all measurements were conducted at room temperature (*T* = 23°C).

### 5.3 Optical Microscope Setup, Image Acquisition, and Light Source

An inverted optical microscope (Olympus IX73) was employed, configured with a dual‐wavelength illumination system to enable controlled photo‐stimulation of the sample. The illumination setup allowed uniform, global exposure of the sample to two discrete wavelengths: 625 nm (red) and 455 nm (blue) and 470 nm (turquoise). Red light (625 nm) does not trigger photo‐isomerization of the photosensitive surfactant, while shorter wavelengths, such as 455 nm (M455L4, Thorlabs company), induce photo‐isomerization depending on their intensity and the selected wavelength [26]. Between the light source and sample, a condenser is installed to render the divergent LED light into a parallel beam to ensure uniform, global illumination. Image acquisition was performed in video recording mode at a frame rate of 30 frames per second using a commercial CMOS camera (Hamamatsu ORCA‐Flash4.0 LT (C11440)). Between the camera and the sample position, a high‐pass filter from the company Thorlabs is installed to avoid undesired reflection of blue light. A scheme of the setup is shown in the Supporting Information in Figure S17.

### 5.4 Nitrogen Sorption Analysis

A microparticle stock suspension (*c*_mass_ = 5 wt.%) containing both non‐porous and porous particles was prepared and combined in the desired mass ratio to yield a final dry sample mass of approximately 100 mg. The resulting particle dispersion was washed twice with Millipore‐grade water to remove residual impurities. Subsequently, the samples were freeze‐dried overnight under high vacuum (<1 mbar) using a Schlenk line. The resulting dried materials were then subjected to further analysis.

#### 5.4.1 Nitrogen Sorption Measurement

Prior to analysis, all dried samples were scaled and loaded into the measurement cells, followed by activation under vacuum at 300°C for 3 h using a commercial BELPREP VAC III sample preparation unit (Microtrac). Nitrogen adsorption–desorption isotherms were subsequently measured using a BELSORP MAX surface area and porosity analyzer (Microtrac). Measurement durations varied between 18 and 43 h, depending on the specific porosity characteristics of each sample. Data is always plotted as the amount of adsorbed nitrogen (volume) against the normalized pressure needed.

### 5.5 Data Acquisitions, Microfluidic Measurements

In a typical measurement, all microparticles were sedimented onto the bottom glass interface of the rectangular microfluidic channel with dimensions of 17 mm in length, 3.8 mm in width, and 0.54 mm in height (µ‐slide^VI^ with a glass bottom cover slip (Ibidi GmbH). The flow was initiated, and video recording was started with a 2‐s delay, using a frame rate of 30 FPS and a resolution of 4012 × 4012 pixels (4K). We applied light illumination with a wavelength of 455 nm with an adjusted power declared in the figure caption or main text. So, in a typical 45 s long measurements, we show moving particles along the flow streamline with the first 5 s without irradiation, followed by 40 s of irradiation of *λ* = 455 nm. For data analysis, the particles crossing in the time range of 10–45 s are used for data analysis (steady state). Each measurement was performed under applied constant pressure‐driven flow using a syringe pump (Ph.D. ultra, Harvard apparatus) with an adjusted volumetric flow rate of 150 µL·min^−1^.

### 5.6 Pumping System Used

For analysis and data library design each measurement was performed under applied constant pressure driven flow using a syringe pump from commercial supplier Ph.D. ultra, Havard apparatus.

To evaluate the impact of flow stability on particle dynamics and measurement accuracy, two peristaltic pumps from different manufacturers were employed:

Peristaltic Pump 1: IPC peristaltic pump, Ismatec

Peristaltic Pump 2 (high precision): Ismatec SA—Vario Pump system

For both configurations and for the syringe pump, the effective volumetric flow rate was set to 200 µL·min^−^^1^, ensuring consistent average throughput across all tests. This controlled setting allowed for a direct comparison of flow‐induced artifacts resulting from each pump's characteristic pulsation profile and pressure stability.

### 5.7 Library Design

For the library, we measured mono‐particle samples with non‐porous (SiO_2_) and porous silica (PSiO_2_; pore diameter:  12 nm) following the same sample preparation steps and measurement procedure as explained above for the binary mixtures. For non‐porous particles, four concentrations were measured (0.1, 0.3, 0.5, and 0.7 mg mL^−1^) and five for porous particles (0.05, 0.1, 0.3, 0.5, and 0.7 mg mL^−1^). From this data, we constructed our data library, which is a database storing all monoparticle measurements with information about their surface chemistry (non‐porous and porous) and the particle concentration. The concentration of the surfactant solution for all samples is set to 2 mM (2 · 10^−3^ mol L^−1^).

### 5.8 Tracking of Particle Motion

We decomposed the recorded video into an image series and calculated the data into binary pixel information (black and white) using the so‐called “thresholding algorithm” from the image software Fiji (Version 1.52u; Java 1.8.0 172). The image series is converted to grayscale. We calculated the particles as objects by their white area in terms of a min‐max interval assumed within the particle range. The identified contours were used to calculate the center of mass for each object to get a local coordinate (x‐y position). From the as‐prepared frame images, object tracking occurred via the retrieval of the minimum distance object within the next frame. This led to the identification of each individual particle trajectory as well as its frame‐to‐frame velocity by multiplying the traveled distance by the framerate and the particle radius. We calculated mean, median velocities observed frame to frame including the standard deviation (see Statistical Analysis Section). The difference in behavior of median and mean thereby allowed detection of potential biases that affected the mean due to extreme outliers. We used a Python executable applying several software libraries: Bokeh, Numpy, OpenCV‐python, Matplotlib, Openpyxl, Pandas, SciPy [24, 26].

The mean frame‐to‐frame particle velocity was calculated similarly to our previous publication [24, 26] with the same set of Python libraries. The software averages over the frame‐to‐frame velocities of all particle *n* (number of particles) in the observed area at the same time *t* using the arithmetic mean:

(7) $${\bar{U}}_{t}=\frac{1}{n}\left(\sum_{i=1}^{n}U_{i}\right)$$

and the corresponding standard deviation *σ* was calculated from the square root of the variance for the sample size of n‐1:

(8) $$\sigma =\sqrt{\frac{1}{n-1}\sum_{n=i}{\left(U_{i}-{\bar{U}}_{t}\right)}^{2}}$$

The average crossing velocity *U*_cross_ was calculated with the arithmetic mean as well, but rather than averaging over an ensemble of particles, we averaged over frame‐to‐frame velocities *U_j_* measured for a single particle crossing the observed area:

(9) $${\bar{U}}_{\mathrm{cross}}=\frac{1}{m}\left(\sum_{j=1}^{m}U_{j}\right)$$

where *m* is the number of frame‐to‐frame velocities determined for the particle. This is the number of frames the particle is inside the observed area minus one.

### 5.9 Outlier Evaluation

As mentioned in Section 4.2, we removed tracks where the path length was smaller than or equal to that of the 0.15 Quantile of the tracked path lengths. This was done with the Python library pandas. Initially, the path length was calculated by subtracting the start frame of each track from its end frame. Then, the 0.15 quantile was determined from the path lengths of all tracks. This was done in three steps. First, the path lengths were sorted from lowest to highest. Second, the quantile is calculated from its position in the sorted column, rounding up:

(10) $$Q_{0.15}=0.15\cdot \left(k+1\right)th\;term$$

where *k* is the number of measured tracks. In the last step, the position is identified with its path length value, giving the quantile. Finally, all path lengths equal to or smaller than the 0.15 quantile are removed from the dataset (pandas dataframe).

Like for the path length, the 0.99 Quantile was determined for the particle area *A*, whose position was calculated as follows:

(11) $$Q_{0.99}=0.99\cdot \left(k+1\right)th\;term$$

After identifying the Quantile for the particle area, *A*, all path lengths equal or larger than the 0.99 quantile were removed from the dataset.

The last parameter we used for outlier removal was the standard deviation of the particle velocity. In this case, all tracks equal or larger than the 0.9 quantile were removed from the dataset.

(12) $$Q_{0.9}=0.9\cdot \left(k+1\right)th\;term$$

To remove the outliers associated with clusters, we searched the dataset for two types of tracks: First, tracks with the same particle area *A* as the slower particles, but with the velocity of the faster particles. For this, we set a maximum area and a minimum velocity; when a particle met both conditions, it was removed from the dataset. The second type are the ones that have the same particle area *A* as the faster particles but with the velocity of the slower particles. These can be found by setting a minimum area and a maximum velocity.

From each of the four types of outliers, we randomly selected representatives to prove that the measures we took were appropriate and targeted the desired type.

### 5.10 Cluster Classification

To cluster the analyte data, with the aim of finding the natural grouping identifying the two particle types, we used an algorithm based on the Gaussian Mixture Model (GMM) provided by the Python library Scikit‐learn. The algorithm is included in the library as a class named Gaussian Mixture. GMM is a probabilistic approach that models each data point as arising from a combination of several Gaussian distributions with unknown parameters. Since the datasets employed in this work are two‐dimensional, with the dimensions' particle area *A* and crossing velocity *U*_cross_, the multidimensional model of the Gaussians is applied:

(13) $$N\left(x|\mu_{i},\Sigma_{i}\right)=\frac{1}{\Sigma_{i}{\left(\sqrt{2\pi }\right)}^{2}}\exp\left(-\frac{1}{2}{\left(x-\mu_{i}\right)}^{T}\Sigma_{i}^{-1}\left(x-\mu_{i}\right)\right)$$

where *x* is a two‐dimensional data point, *µ*_k_ is the mean and Σ_k_ the covariance matrix of the Gaussian. The number of Gaussians in the Gaussian mixture is determined by the number of clusters. Since the analyte is a binary mixture the number was set to two. With the known number of components, the technique of expectation maximization (EM) is employed to maximize the following likelihood function:

(14) $$L\left(\phi ,\mu ,\Sigma |X\right)=\prod_{n=1}^{N}\sum_{i=1}^{K}\phi_{i}\cdot N\left(x_{n}|\mu_{i},\Sigma_{i}\right)$$

where ϕ_*i*_ is the mixture component weight of the *i*‐th Gaussian and *X* denotes the entire dataset. As soon as a local maximum or a saddle point of the likelihood function is reached, the probability for each datapoint *x* belonging to the two Gaussians (i.e., clusters) is calculated by the following equation:

(15) $$p\left(C_{i}|x\right)=\frac{\phi_{i}N\left(x|\mu_{i},\Sigma_{i}\right)}{\sum_{j=1}^{K}\phi_{j}N\left(x|\mu_{j},\Sigma_{j}\right)}$$

where *C*_i_ stands for the *i*‐th Gaussian. A datapoint is assigned to the cluster where it belongs with the highest likelihood. In this way, all datapoints are labeled either with Cluster 0 or Cluster 1. A detailed explanation of the Gaussian Mixture Model and the expectation maximisation technique is given in Section S3.

### 5.11 Quantile Calculations

As already stated, the matching of the clusters with the data library was achieved by Q‐Q plots. For each cluster, Q‐Q plots were made with each entry of the library. The procedure was the same for every plot: First, the crossing velocities ***U***_cross_ of the library dataset and the cluster dataset were sorted from smallest to largest value. Then the sample size regarding the crossing velocities ***U***_cross_ is determined for both datasets. With this information, a number of quantiles that is equal to the sample size M of the smaller dataset is determined for the larger sample. Accordingly, the smallest quantile is the 1/M‐quantile and the largest is the 1‐Quantile. The p‐quantile is given by:

(16) $$Q_{p}=p\cdot \left(K+1\right)th\;term$$

where *K* is the number of data points of the larger sample. The next step depends on whether the cluster or the library dataset had the larger sample size. When that of the cluster was the larger one, the quantiles of the cluster were plotted on the *x*‐axis, and the ordered values of the library on the ***y***‐axis. If it were the other way around, the ordered values of the cluster were plotted on the ***x***‐axis, and the quantiles of the library on the ***y***‐axis. Both datasets are considered similarly distributed if they lie on a straight line given by the identity function.

(17) $$f\left(x\right)=x$$

To quantify the similarity of the datasets, the coefficient of determination *R* ^2^ was calculated from the Q‐Q plot and the identity function.

When all Q‐Q plots were performed, the *R* ^2^ values were compared. The best match between cluster and library was identified by finding the *R* ^2^ values closest to one. For more details on the Q‐Q plot, see Section S5 (Supporting Information).

## Author Contributions

M.B. designed and supervised the project and wrote the manuscript. F.R. performed LDV experiments and analysis and wrote the manuscript. D.V. and I. M. supported and assisted in LDV measurements. N.L. synthesized the photosensitive surfactant. M.R. and F.S. prepared samples for nitrogen sorption analysis. A.N. and A.T. performed nitrogen sorption analysis. A.T. and S.S. wrote the manuscript. All the authors were involved in the preparation of the manuscript. All the authors have read and approved the final manuscript.

## Conflicts of Interest

The authors declare no conflicts of interest.

## Supporting information




**Supporting File 1**: smtd70568‐sup‐0001‐SuppMat.docx.

**Supporting File 2**: smtd70568‐sup‐0002‐VideoS1.mov.

**Supporting File 3**: smtd70568‐sup‐0003‐VideoS2.mov.

**Supporting File 4**: smtd70568‐sup‐0004‐VideoS3.mov.

**Supporting File 5**: smtd70568‐sup‐0005‐VideoS4.mov.

**Supporting File 6**: smtd70568‐sup‐0006‐VideoS5.mov.

**Supporting File 7**: smtd70568‐sup‐0007‐VideoS6.mov.

## Data Availability

The data that support the findings of this study are available from the corresponding author upon reasonable request.
