# Supplementary material for: Particle‐Based Detection of Surface Chemistry via Optical Microscopy—Integrating Microfluidics, Light‐Induced Activity of Colloids and Data Science
Source: Small Methods. 2026 Feb 27;10(6):e02329. doi: 10.1002/smtd.202502329 (PMC13010204; doi:10.1002/smtd.202502329)
Supplement: Supplementary file 1 — Supporting File 1: smtd70568‐sup‐0001‐SuppMat.docx. [file SMTD-10-e02329-s003.docx]

Supporting information

Particle Based Detection of Surface Chemistry via Optical Microscopy and Microfluidics

Fabian Rohne,^1^ Daniela Vasquez Muñoz,^1^ Isabel Meier,^1^ Anne Nitschke,^3^ Florian Schmitt, Nino Lomadze^1^ Martin Reifarth,^2^ Andreas Taubert,^3^ Svetlana Santer,^1^ Marek Bekir,^1*^

Fabian Rohne,^1^ Daniela Vasquez Muñoz,^1^ Isabel Meier,^1^ Nino Lomadze^1^ Svetlana Santer,^1^ Marek Bekir,^1^

*University Potsdam, Institute of Physics and Astronomy, Karl-Liebknecht-Str. 24-25, D-14476 Potsdam*

M. Reifarth,^2^ F. Schmitt,^2^

*University Potsdam, Institute of Chemistry, Karl-Liebknecht-Str. 24-25, D-14476 Potsdam*

*Fraunhofer Institute of Applied Polymer Research, Geiselbergstr. 69, D-14476 Potsdam*.

A. Nitschke,^3^ A. Taubert^3^

*University Potsdam, Institute of Chemistry, Karl-Liebknecht-Str. 24-25, D-14476 Potsdam*

# Data display for the choice of Gaussian Mixture Model as Clustering algorithm


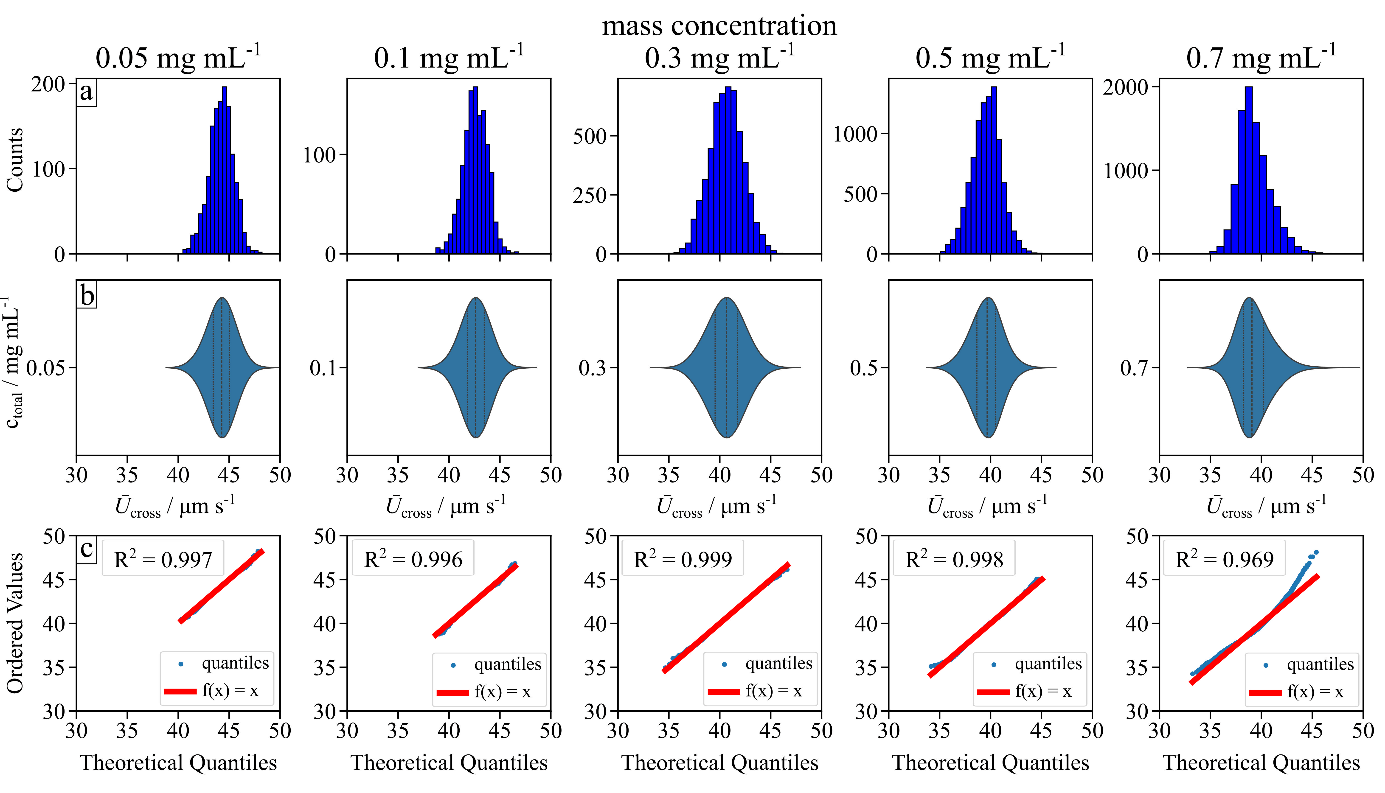


**Figure S1**. Visualization of particle velocity distributions under light illumination for varying concentrations of pure porous silica particles (PSiO_2_). (a) histogram, (b) violin-, and (c) Q-Q plot of the library datasets of porous silica (PSiO_2_) for all total mass concentrations 𝑐_total_.


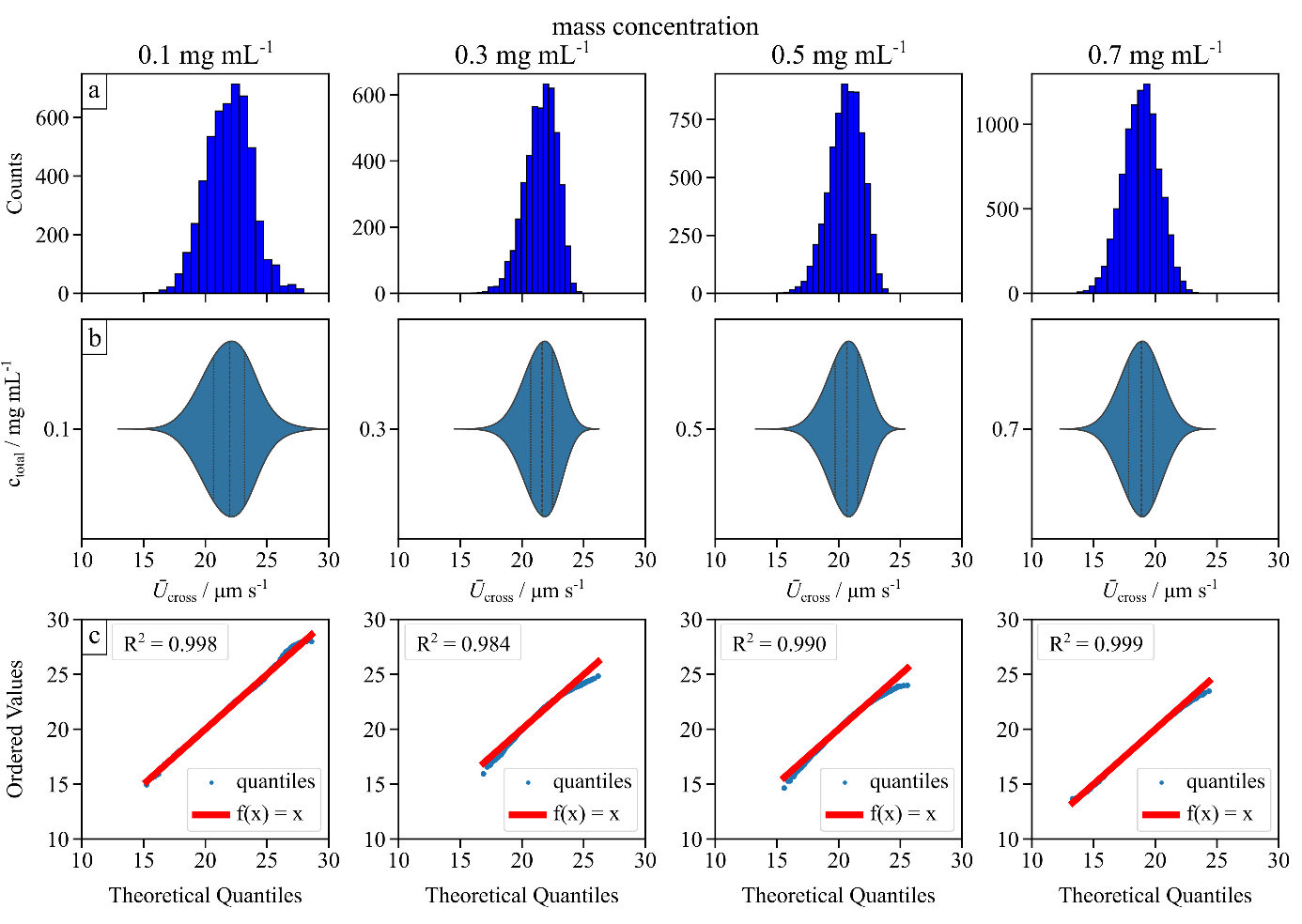


**Figure S2**. Visualization of particle velocity distributions under light illumination for varying concentrations of pure non-porous silica particles (SiO_2_). (a) histogram, (b) violin-, and (c) Q-Q plot of the library datasets of non-porous silica (PSiO_2_) for all total mass concentrations 𝑐_total_.

# Cluster misidentification on *U*_cross_ data sets via Q-Q plots – no Light

Demonstration of miss-classification if light is switched off. Although the sample contains the mixture of SiO_2_ and PSiO_2_ the algorithm only yields two classified SiO_2_ particles with *R*^2^ of -0.82 or 0.52.


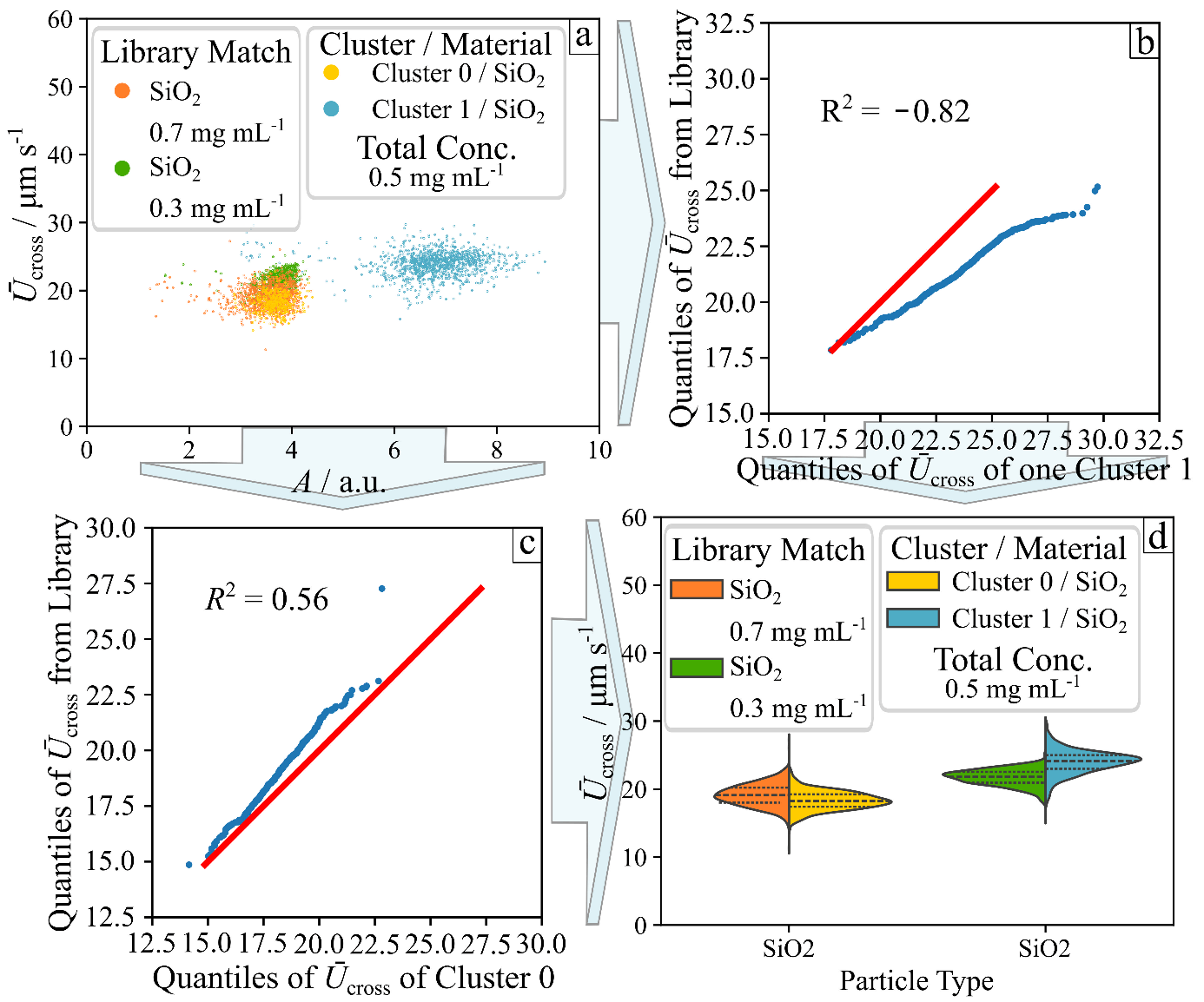


**Figure S3**. Example display of **data miss-identification** by quantifying by the best *R*^2^ value from the quantiles of the velocity *U*_cross_ of library data against one Cluster. (a) Clusters of measured data points library (same data as in Figure 3e). (b,c) Example fit of quantiles (d) Calculated violin plot distributions of velocity *U*_cross_ classified by particles – library data and measurement. Straight line illustrates the average value of *U*_cross_, dotted line visualizes the 25 %- and 75 %-percentile of $\bar{U}_{\mathrm{cross}}$.

# Clustering with Gaussian Mixture Model

Cluster analysis is a category of unsupervised learning techniques that allow us to discover hidden structures in data where we do not know the right answer upfront. In contrast to supervised learning there is no training data available where labels are already assigned and thus the searched structures are not known. Accordingly, the goal of clustering is to find a natural grouping in data so that items in the same cluster are more similar to each other than to those from different clusters.^[^^[[1]](#endnote-1)]^

These hidden groupings can have various shapes and structures inside the data. To meet this challenge a variety of clustering algorithms were developed. In this work the Gaussian Mixture Model (GMM) was used. GMM is a probabilistic approach that models each data point as arising from a combination of several Gaussian distributions with unknown parameters. Unlike hard clustering methods such as the well-known *K*-Means, which rigidly assigns each point to a single cluster based on proximity to the nearest centroid, an assumption that may oversimplify real-world data, GMMs enable soft clustering. This means each data point can belong to multiple clusters simultaneously, with degrees of membership represented as probabilities.^[^^[[2]](#endnote-2)]^

A Gaussian mixture model is parametrized by two types of values, the mixture component weights, and the component means and covariance. All K components of the Gaussian mixture are either normal distributions if the data is one-dimensional or multivariate normal distribution if the data is multi-dimensional. In the univariate case the *k*^th^ component has a mean of $\mu_{k}$ and variance of $\sigma_{k}$, and a mean $\boldsymbol{\mu}_{k}$ and covariance matrix $\Sigma_{k}$ for the multivariate case. The mixture component weights are defined as $\phi_{k}$ for component $C_{k}$ with the constrain:^[[3]](#endnote-3)^

| $\sum_{i=1}^{K} \phi_{i}=1$, | Eq. S1 |
| --- | --- |

which ensures that the probability distribution normalizes to 1. If the weights $\phi_{k}$ are not learned from data, they act as prior probabilities for selecting a component—meaning the probability that a data point ***x*** is generated by component $C_{k}$ is simply $\phi_{k}$. When the weights are learned instead, they represent the posterior probabilities, reflecting the likelihood of each component given the observed data. In this case the probability that data point is generated under the condition that it is generated by the *k*^th^ component is given by:^[3]^

| $\mathcal{N}\left( x \mid\mu_{i},\sigma_{i} \right)=\frac{1}{\sigma_{k}\sqrt{2\pi}}\exp\left( -\frac{\left( x-\mu_{i} \right)^{2}}{2\sigma_{i}^{2}} \right)$, | Eq. S2 |
| --- | --- |

for a one-dimensional model and

| $\mathcal{N}\left( \boldsymbol{x} \mid\boldsymbol{\mu}_{i},\Sigma_{i} \right)=\frac{1}{\Sigma_{i}\left( \sqrt{2\pi} \right)^{K}}\exp\left( -\frac{1}{2}\left( \boldsymbol{x}-\boldsymbol{\mu}_{i} \right)^{\text{T}}\Sigma_{i}^{-1}\left( \boldsymbol{x}-\boldsymbol{\mu}_{i} \right) \right)$, | Eq. S3 |
| --- | --- |

for a multidimensional model. Accordingly, the probability that a data point $x$ is generated by any of the gaussians is:

| $p\left( x \right)=\sum_{i=1}^{K} \phi_{i}\mathcal{N}\left( x \mid\mu_{i},\sigma_{i} \right)$ , | Eq. S4 |
| --- | --- |

for a one-dimensional model and

| $p\left( \boldsymbol{x} \right)=\sum_{i=1}^{K} \phi_{i}\mathcal{N}\left( \boldsymbol{x} \mid\boldsymbol{\mu}_{i},\Sigma_{i} \right)$ , | Eq. S5 |
| --- | --- |

for a multidimensional model. Form $\mathcal{N}\left( x \mid\mu_{i},\sigma_{i} \right)$ and p(x) the probability that a data point $x$ belongs to cluster $C_{k}$​ can be calculated:

| $p\left( C_{k} \mid x \right)=\frac{p\left( x\cap C_{k} \right)}{p\left( x \right)}$, | Eq. S6 |
| --- | --- |

using Bayes’ theorem, the conditional probability can be rewritten

| $p\left( C_{i} \mid x \right)=\frac{p\left( C_{i} \right)p\left( x \mid C_{i} \right)}{\sum_{j=1}^{K} p\left( C_{j} \right)p\left( x \mid C_{j} \right)}=\frac{\phi_{i}\mathcal{N}\left( x \mid\mu_{i},\sigma_{i} \right)}{\sum_{j=1}^{K} \phi_{j}\mathcal{N}\left( x \mid\mu_{j},\sigma_{j} \right)}$, | Eq. S7 |
| --- | --- |

and analogous for a multidimensional model

| $p\left( C_{i} \mid\boldsymbol{x} \right)=\frac{\phi_{i}\mathcal{N}\left( \boldsymbol{x} \mid\boldsymbol{\mu}_{i},\Sigma_{i} \right)}{\sum_{j=1}^{K} \phi_{j}\mathcal{N}\left( \boldsymbol{x} \mid\boldsymbol{\mu}_{j},\Sigma_{j} \right)}$ . | Eq. S8 |
| --- | --- |

This probability plays a key role in learning the model and will later determine the cluster to which a data point is assigned to.

The gaussian mixture model is learned by estimating the mixture model’s parameters. If the number of components is known expectation maximation (EM) is the most common technique. It is a computational method used to perform maximum likelihood estimation, particularly effective when the model parameters can be updated using closed-form expressions. EM operates as an iterative algorithm and has the advantageous trait that the likelihood of the observed data consistently improves with each step. As a result, the algorithm is guaranteed to converge to a local maximum or a saddle point of the likelihood function.^[3]^

EM for mixture models involves two key phases carried out iteratively:

In the E-Step (Expectation Step), the model parameters $\phi_{k},\mu_{k}$ and $\sigma_{k}$ are given and the expectation that a data point $x$ belongs to component $C_{k}$​ are calculated for each data point $x_{i}\in X$ using equation Eq. S7 or Eq. S8 depending on the dimensionality. Prior to the first E-step an initialization step is made, which assigns model parameters to reasonable values based on the data.

The second step, the M-Step (Maximization Step)**,** uses the assignments calculated during the E-step, this phase updates the model parameters $\phi_{k}$, $\mu_{k}$, and $\sigma_{k}$ to maximize the likelihood of the observed data.

This cycle of E and M steps is repeated until the algorithm converges to a set of parameters that locally maximizes the likelihood function:^3^

| $\mathcal{L}\left( \phi,\mu,\sigma\mid X \right)=\prod_{n=1}^{N} \sum_{i=1}^{K} \phi_{i}\cdot\mathcal{N}\left( x_{n} \mid\mu_{i},\sigma_{i} \right)$ , | Eq. S9 |
| --- | --- |

for a one-dimensional model and

| $\mathcal{L}\left( \phi,\boldsymbol{\mu},\Sigma\mid X \right)=\prod_{n=1}^{N} \sum_{i=1}^{K} \phi_{i}\cdot\mathcal{N}\left( \boldsymbol{x}_{n} \mid\boldsymbol{\mu}_{i},\Sigma_{i} \right)$ , | Eq. S10 |
| --- | --- |

for a multidimensional model.

Summing up, the intuition behind EM lies in alternating assumptions. If we knew which component $C_{k}$ generated each point $x_{i}$, estimating the parameters $\phi_{k}$, $\mu_{k}$, and $\sigma_{k}$ would be straightforward. Conversely, if we had accurate parameters, it would be easy to infer the probability that a given $x_{i}$ was generated by component $C_{k}$. The E-step assumes the parameters are fixed to infer assignments, while the M-step assumes the assignments are fixed to update the parameters. This back-and-forth optimization efficiently leads to a maximum likelihood estimate. When a local maximum of the likelihood function is found the cluster assignment for each data point is determined by its most likely component assignment, from equation Eq. S7 or Eq. S8 depending on the dimensionality.^[3]^


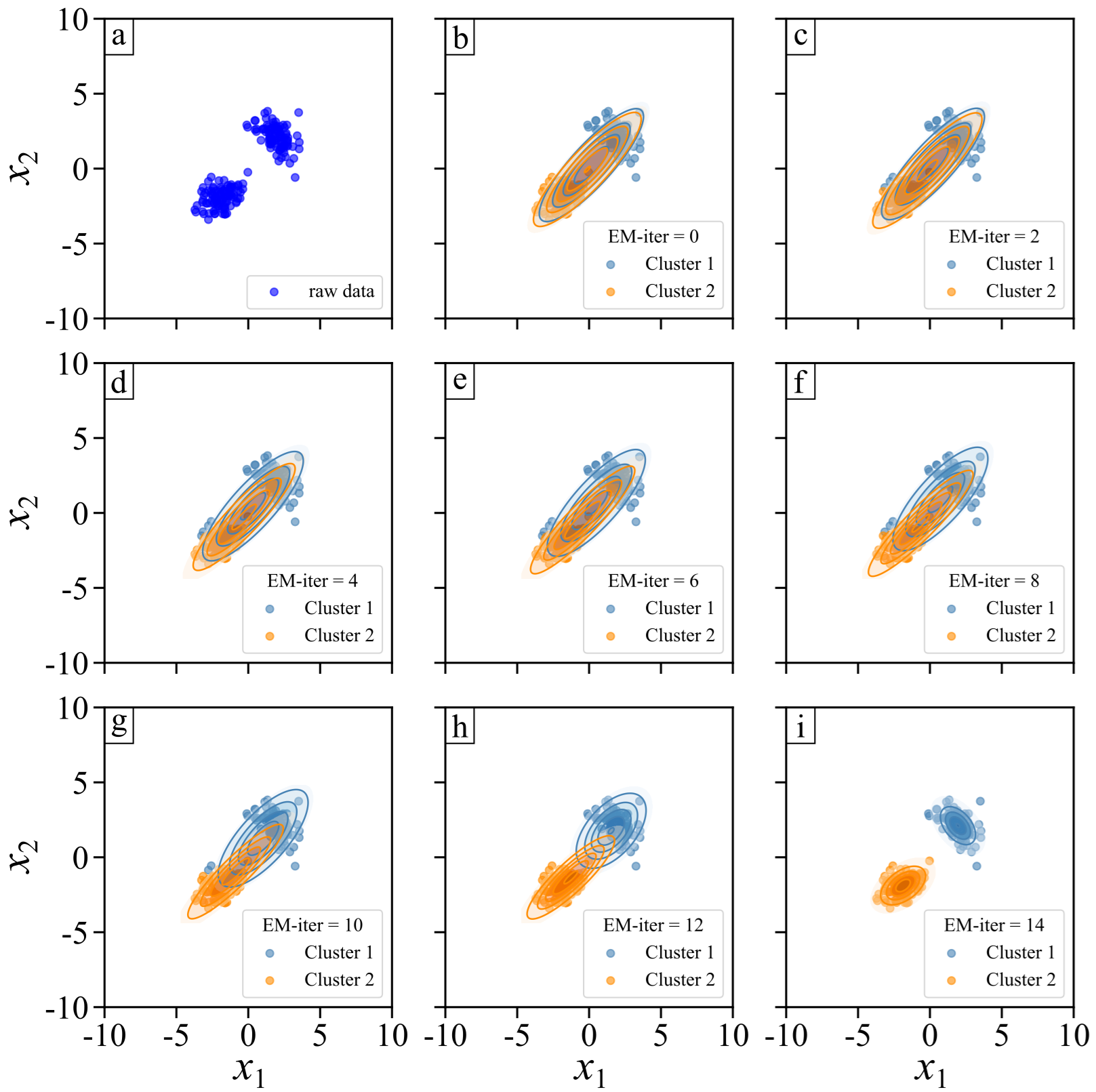
**Figure S4**. Graphical representation of the EM algorithm applied to (a) a simple example of data points generated by two Gaussians. (b) The initial step, where the parameters for the Gaussian are randomly guessed. Contour lines depict the two-dimensional Gaussians. The component assignments are indicated by their color (Cluster 1: blue, Cluster 2: orange. The results of the EM algorithm are shown after (c) 2 steps, (d) 4 steps, (e) 6 steps, (f) 8 steps, (g) 10 steps, (h) 12 steps, and (i) 14 steps.

# Violine plots

A violine plot combines a Box-Plot with a Kernal-Density-Estimation (KDE). Thus, it is a useful tool for graphical comparison of statistics of data sets.

Box-Plots offer a quick option to visualize the distribution of data based on percentiles. In a dataset, the P%-percentile is defined such that at least P% of the values are less than or equal to this value, and at least (100 – P)% of the values are greater than or equal to it. A commonly used measure of statistical dispersion is the difference between the 25%- and the 75%-percentile the so-called interquartile Range (IQR). Together with the 50%-percentile, which is the median, the IQR is the heart of a Box-Plot. The upper line of the rectangular box is the 75%-percentile and the lower one is the 25%-percentile. The thick line inside the box is the median. Accordingly, the Box represents 50% of the data points. In case the median is not in the middle of the Box the data set is skewed. This can be verified by looking at Figure S4. The Box-Plot with data generated by a normal distribution, which is known to be symmetric, shows median in the middle of the box. In contrast the Box-Plot with data generated by a normal distribution, which is skewed, shows a median closer to the 25%- percentile. To include the tails of the data the Box-Plot includes vertical lines called Whisker. The upper line of the Whisker is either the sample maximum or the 75%-percentile plus 1.5 times the IQR when this value is smaller than the maximum. Following the same idea the lower line of the Whisker is either the sample minimum or the 25%-percentile minus 1.5 times the IQR when this value is larger than the minimum. Data points not included in the Whiskers are marked with black circles. Comparing the Box-Plot of data generated by a normal distribution with that generated by a Laplace distribution indicates that the tales of a Laplace distribution are much more pronounced than those of a normal distribution (see Figure S5a).

In violin plots the Box-Plots are often simplified as shown in Figure S5b. In this work there are reduced to three horizontal lines, the mean in the middle and the upper and lower line for the 25%- and the 75%-percentile respectively.

All three are inside the probability density function estimated by KDE (see Figure S5c). Based on a finite data sample this non-parametric method can estimate the probability density function of a random variable. Unlike histograms, which rely on discrete bins, KDE produces a smooth and continuous curve that reflects the underlying distribution more naturally. The core idea behind KDE is to place a smooth, symmetric function—called a kernel—at each data point and then sum these functions to obtain the overall density estimate. Given a random variable *X* with n observations the KDE starts by centering a kernel function *K* at each data point $x_{i}$ (See Figure S5). A commonly used kernel, which ensures that the contribution of each data point is smoothly distributed around its location, is the Gaussian function, defined as

| $K\left( u \right)=\frac{1}{\sigma\sqrt{2\pi}}e^{-\frac{u^{2}}{{2\sigma}^{2}}}$, | Eq. S11 |
| --- | --- |

where *u* is the scaled distance $\left( x-x_{i} \right)/h$. The bandwidth *h* determines the width of the kernel and thereby controls the smoothness of the resulting density estimate. Accordingly, a smaller bandwidth leads to an estimate that closely follows the data, while a larger bandwidth generates a smoother curve that may obscure finer details. To get the estimated density at a position *x* it has to be summed over the contributions of all kernels, also called bumps, at this position:

| $\hat{f}_{h}\left( x \right)=\frac{1}{nh}\sum_{i=1}^{n} K\left( \frac{x-x_{i}}{h} \right)$, | Eq. S12 |
| --- | --- |

where the factor $1/\left( nh \right)$ ensures that the density estimation is normalized. The function gives a smooth curve which is the estimated probability density function of the data set reflecting the concentration of data points (See Figure S5c, KDE as blue plot).


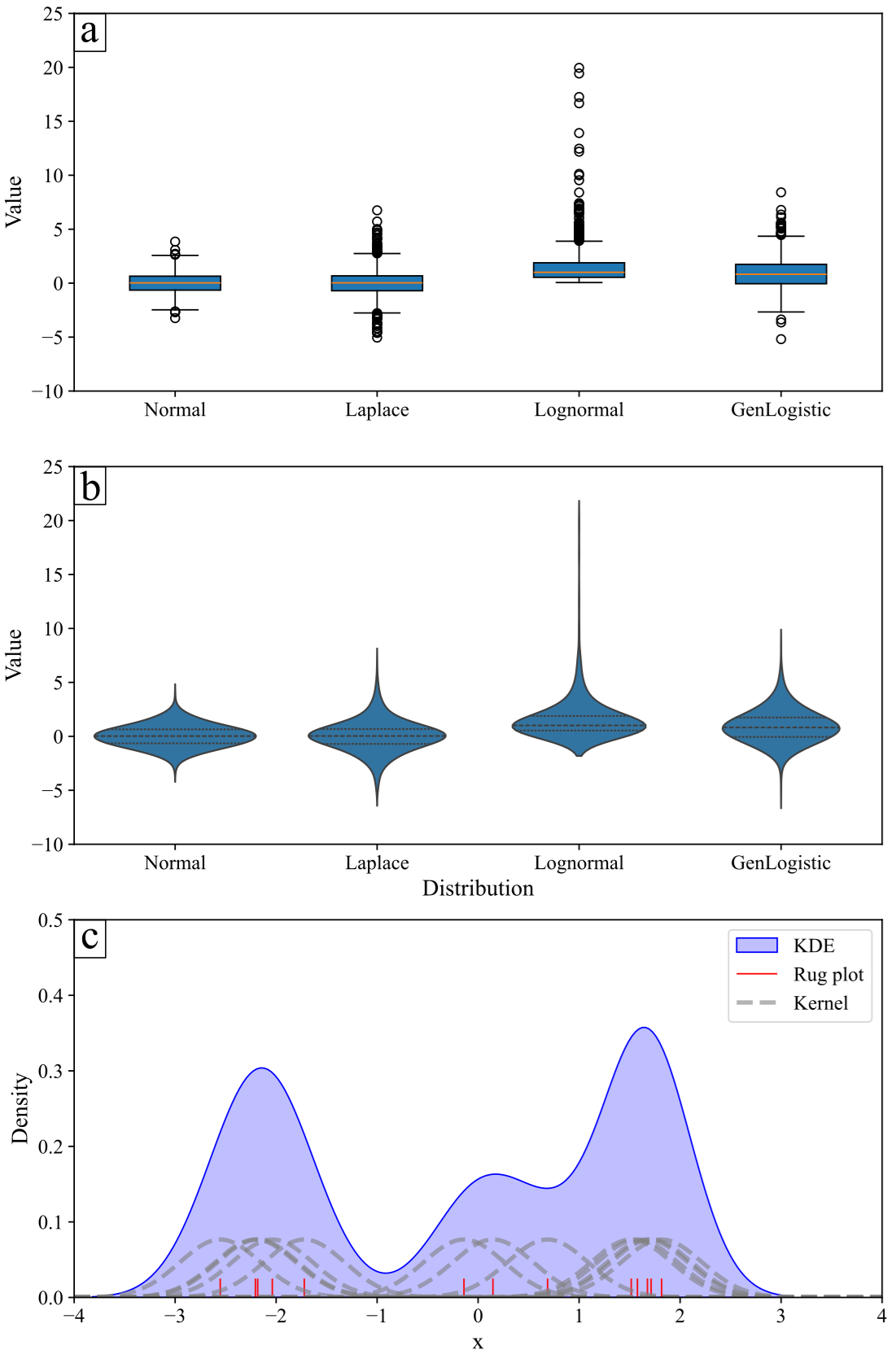


**Figure S5**. (a) box plots of data generated by a normal, Laplace, log-normal, or generalized-logistic distribution. (b) Violin plots of the same data that was used for box plots in (a). (c) Example for a kernel density estimation (blue line) where the red rugs are the data points and the grey dashed lines are the kernels centered around each data point.

# Quantile-Quantile Plot


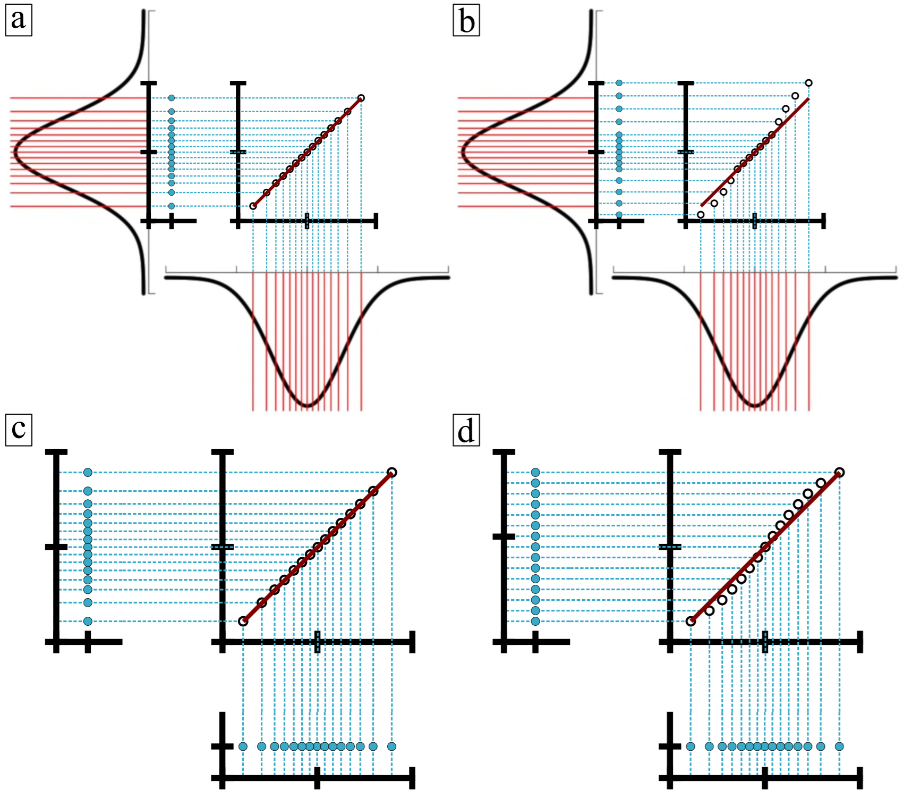


**Figure S6**. Schematic representation of quantile-quantile plots between: (a) a dataset following a Gaussian distribution and the quantiles of the generating Gaussian distribution, (b) a dataset where the central portion follows a Gaussian distribution but the tails follow uniform distributions and the quantiles of the Gaussian distribution generating the central portion, (c) two datasets generated by the same Gaussian distribution, (d) a dataset following a Gaussian and one following a uniform distribution. For simplicity, the number of both datasets is the same.

A quantile-quantile plot is a graphical representation used to check whether a data set follows a certain probability density function or to compare the distribution of two data sets.

For example, to show that a data set follows a gaussian distribution using a quantile-quantile plot we first order the data set in ascending order. To later compute the quantiles. Then the mean

| $\mu=\frac{1}{N}\left( \sum_{i=1}^{N} x_{i} \right)$, | Eq. S13 |
| --- | --- |

and standard deviation

| $\sigma=\sqrt{\frac{1}{N-1}\sum_{i=1}^{N} \left( x_{i}-\mu\right)^{2}}$ . | Eq. S14 |
| --- | --- |

of the data set are determined to calculate the probability density function for the gaussian distribution:

| $f(x)=\frac{1}{\sigma\sqrt{2\pi}}\exp\left( -\frac{\left( x-\mu\right)^{2}}{2\sigma^{2}} \right)$, | Eq. S15 |
| --- | --- |

Now the quantiles of the gaussian distribution are computed. The number of quantiles is given by the size of the data set with N data points. Accordingly, the smallest quantile is that of the smallest value in the data set which is the 1/N-Quantile. The largest quantile that can be determined is the N-1/N-Quantile. For a gaussian distribution the quantiles can be calculated using the inverse cumulative distribution function:

| $x = F^{-1}\left( p \mid\mu,\sigma\right)=\mu+\sigma\cdot\Phi^{-1}\left( p \right)$, | Eq. S16 |
| --- | --- |

where $\Phi^{-1}\left( p \right)$ the standard normal inverse CDF often approximated using the inverse error function:

| $\Phi^{-1}\left( p \right)=\sqrt{2} \mathrm{erf}^{-1}\left( 2p-1 \right)$, | Eq. S17 |
| --- | --- |

Since a p-quantile divides a probability measure into a left part with the probability *p* and a right part with the probability 1-*p*. Each of the N *p*-quantiles can be easily determined by inserting *p* into Eq. S16.

After the quantiles are calculated the sorted data is plotted on the *y*-axis against the corresponding theoretical quantiles on the *x*-axis. Each data point (*x*,*y*) is a pair of observed and expected value. If the point lay on a linear function with slope 1 and a y-intercept at the coordinate origin it can be assumed that the data set follows a gaussian distribution with the calculated $\mu$ and $\sigma$. This interpretation holds because observed and expected values are equal. In the case that no straight line is given the data does not follow the gaussian distribution. Another possible observation could be that the data is locally gaussian distributed but for instance the tails are not.

However, quantile-quantile plot can also be used to compare the distribution of two data sets. For this purpose both samples are ordered from smallest to largest value. Then a number of quantiles that is equal to the sample size *M* of the smaller data set is determined for the larger sample. Here the smallest quantile is the 1/*M*-quantile and the largest is the 1-Quantile. The *p*-quantile is given by:

| $Q_{p}=p\cdot\left( K+1 \right)th term$, | Eq. S18 |
| --- | --- |

where *K* is the number of data points of the larger sample. In the next step the sorted values of the smaller sample are plotted against the quantiles of the larger sample. Like in the quantile-quantile plot with the Gaussian distribution, the data sets are similarly distributed if the lay on a straight line. Maximum consistency between the data sets is given when all points lay on a linear function with a slope of 1 and a *y*-intercept at the coordinate origin. Accordingly, the coefficient of determination $R^{2}$ calculated from the quantile quantile plot and the identity function gives a measure for the similarity of both data sets. The closer $R^{2}$ value is to 1 the higher the similarity.

# Description of the Quantification Methodes

The classification accuracy compares the fractions that were calculated from the number of cluster trajectories with the theoretical number fractions determined from the mass ratios given by the sample preparation. How the ratios are obtained from the clustering results is already explained in Section 2.3 in the main text. In this section equations and explanations for the calculation of the theoretical number ratio and the classification accuracy are given.

To get the theoretical number ratio we first calculated the adjusted mass concentration $c_{p}$ of each particle type in the mixture, where p is the index for the particle type either PSiO_2_ or SiO_2_.

| $c_{p}=c_{\text{total}}\cdot M_{p}$*,* | Eq. S19 |
| --- | --- |

where $M_{p}$ is the adjusted mass fraction for the respective particle type and the total concentration $c_{total}$. The mass fraction can be easily computed from the mass ratios by dividing the parts of the respective particle $P_{p}$ with the total number of parts $P_{\text{total}}$:

| $M_{\text{p}}=\frac{P_{\text{p}}}{P_{\text{total}}}$*,* | Eq. S20 |
| --- | --- |

From the adjusted mass the theoretical number of particles per volume for a species $N_{p}$ is calculated by dividing it through the mass of one particle $m_{p}$:

| $N_{\text{p}}=\frac{c_{\text{p}}}{m_{\text{p}}}$*,* | Eq. S21 |
| --- | --- |

The mass of one particle $m_{p}$ was calculated assuming that the particles are perfect spheres so the volume $V_{\text{S}}$ is given by:

| $V_{\text{p}}=\frac{4}{3}\pi a_{\text{p}}^{3}$*,* | Eq. S22 |
| --- | --- |

where $a_{\text{p}}$ is the radius which was the same for both particles 2.5 $\mu$m. With the given particle material densities $\rho_{\text{p}}$ (~1.8 g/cm^3^ (PSiO_2_), 1.85 g/cm^3^ (SiO_2_)) we computed the mass per particle:

| $m_{\text{p}}=\rho_{\text{p}}V_{\text{p}}$*,* | Eq. S23 |
| --- | --- |

The theoretical number fraction $n_{theo,p}$ for one species p is determined by dividing the theoretical number $N_{p}$ of that species by the sum of the theoretical number of both species

| $n_{theo,p}=\frac{N_{\text{p}}}{N_{\text{PSi}\text{O}_{\text{2}}}+N_{\text{Si}\text{O}_{\text{2}}}}=\frac{N_{\text{p}}}{N_{\text{total}}}$*,* | Eq. S24 |
| --- | --- |

where $N_{\text{total}}$ is the total theoretical number of particles per volume.

From that theoretical number fraction $n_{theo,p}$ and number fraction $n_{clu,p}$ calculated from the cluster trajectories the deviation of fractions was computed for both species:

| $\text{deviation}_{\text{p}}=\frac{n_{\text{clu,p}}}{n_{\text{theo,p}}}-1$*,* | Eq. S25 |
| --- | --- |

Accordingly, each analyte yields two deviations from which we calculated an average deviation per analyte.

| ${average dev.}_{c,r}=\frac{\mathrm{deviation}_{\text{PSi}\text{O}_{\text{2}}}+\mathrm{deviation}_{\text{Si}\text{O}_{\text{2}}}}{2}$*,* | Eq. S26 |
| --- | --- |

where *c* denotes the total mass concentration and *r* the adjusted mass ratio. The classification accuracy was calculated for all samples with the same total mass concentration by averaging over the classification accuracies of the three adjusted mass rations differing for the total mass concentration:

| ${average dev.}_{c}=\frac{{\mathrm{average} dev.}_{\text{c,r1}}+{\mathrm{average} dev.}_{\text{c,r2}}+{\mathrm{average} dev.}_{\text{c,r3}}}{3}$*,* | Eq. S27 |
| --- | --- |

Furthermore, average classification accuracy for all measured analytes is determined by averaging over the classification accuracy of all analytes

| $aver. class.accu.=\frac{{average dev.}_{0.1}+{average dev.}_{0.5}+{average dev.}_{0.7}}{3}$*,* | Eq. S28 |
| --- | --- |


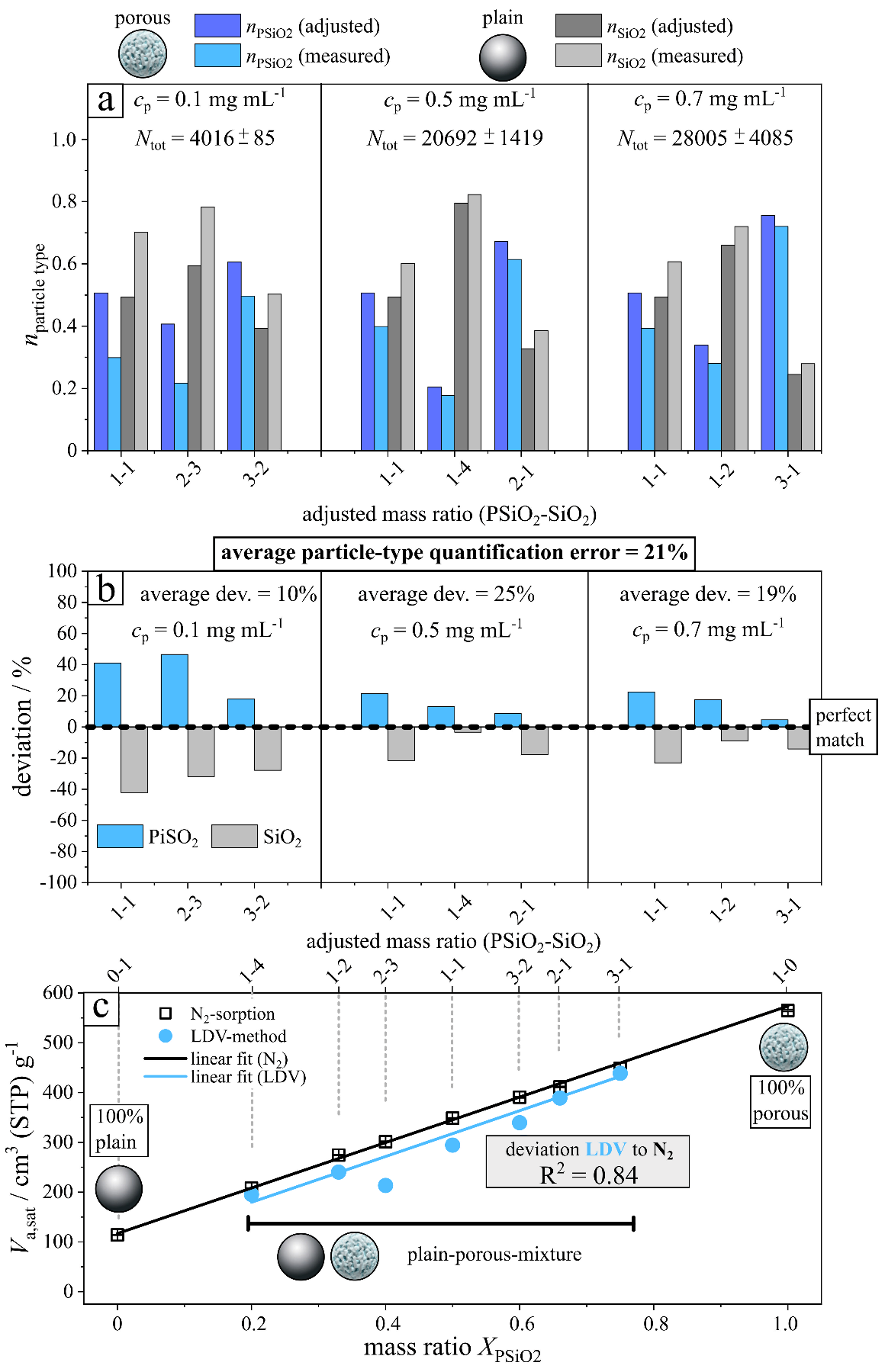


**Figure S7**. Display of quantification accuracy according to quantification method (ii). (a) calculated number fraction *n* from sample adjustment and measurement of crossing porous and plain silica microparticles. Data presented in *n* versus theoretical mass sample ratio and is further classified in total mass concentration. The total mass concentration and corresponding total number of particles is given in the figure. (b) deviation from the theoretical value for the same classified sample ratio and total sample concentration. (c) measured and approximated of adsorbed saturation volume for the same data set.

# Raw Nitrogen Isotherms


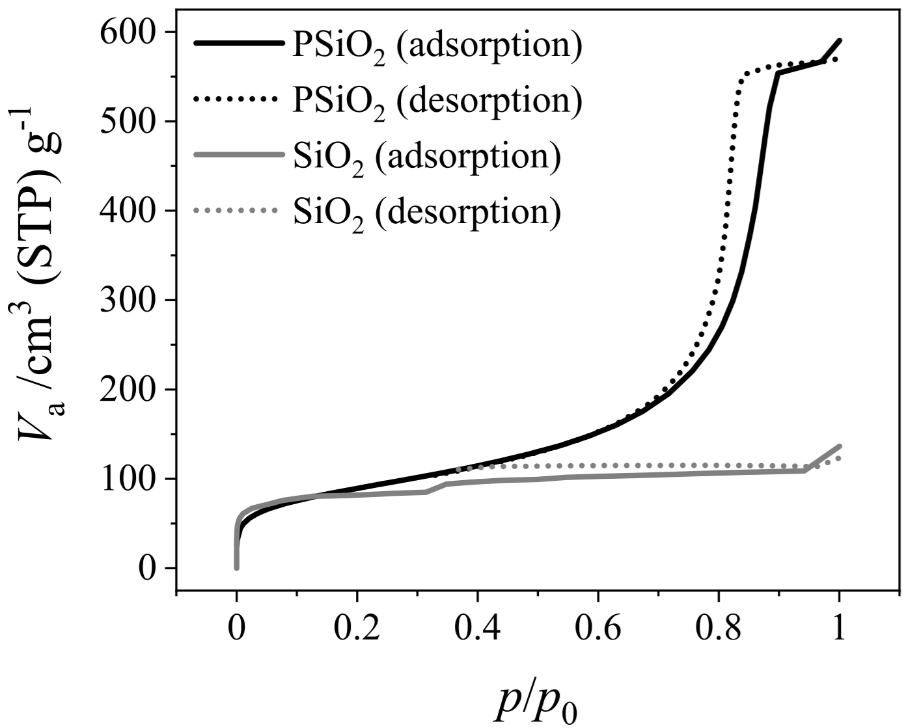


**Figure S8**. Nitrogen (a) adsorption and desorption isotherm of pure non-porous (SiO_2_) and pure porous (PSiO_2_) particles.


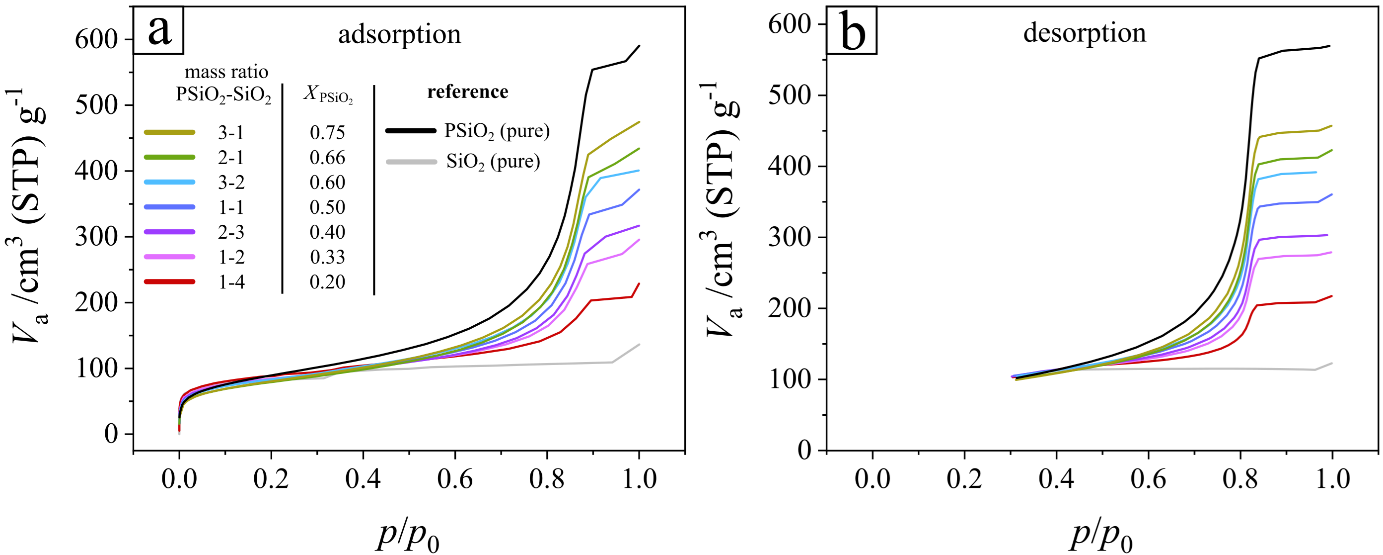


**Figure S9**. Nitrogen (a) adsorption and (b) desorption isotherm. Plotted is the nitrogen volume adsorbed as function of applied nitrogen pressure. Data classified by different particle ratios. For the analysis in the main article the data for saturation volume *V*_a,sat_ is taken from panel (b), averaged from the range 0.8–1.0 and plotted in Figure 4 main article.

# Concentration Dependence and Stability of the crossing velocity $\boldsymbol{U}_{\mathbf{cross}}$


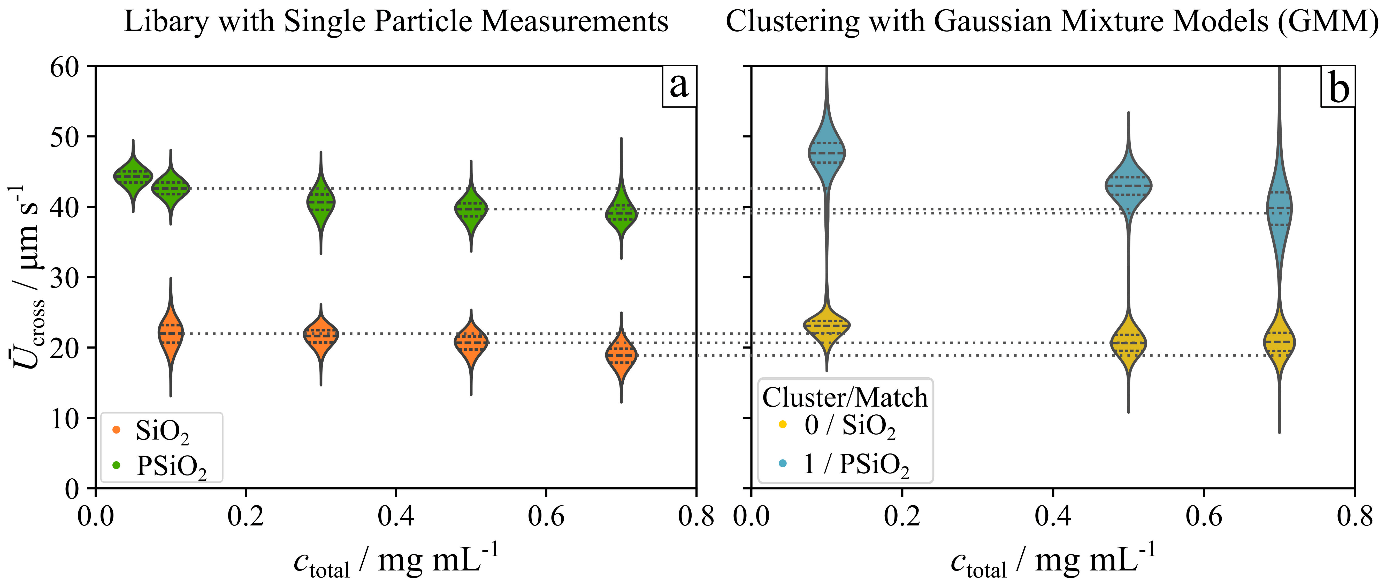


**Figure S10**. Violin plots of (a) the library data for porous and non-porous particles (PSiO_2_ and SiO_2_) and (b) the clustered analyte data for the three mass concentrations and same ratio 1:1. The dotted lines originate from the medians of the library datasets and end at the position where they hit the cluster corresponding to the same total mass concentration

In the single particle measurements, we see two clear trends for the dependents of the crossing velocity on concentration for the two species. For the slower non-porous particles, dependence can be described as a concave function since for small concentrations the $U_{\mathrm{cross}}$ seems to be constant and then decreases with increasing concentration for concentrations above 0.3 mg mL^–1^. For the faster porous particles, the behavior of $U_{\mathrm{cross}}$ is better described by a convex function since it decreases with increasing concentration, but the slope is decreasing in same direction. For high concentrations equal and above 0.5 mg/mL a plateau is reached.

While the crossing velocity decreases with increasing concentration as well, no match of velocities is found between library measurements and clusters from the analyte measurements for same total mass concentrations. As explained in the main text, this is a consequence of the mixing of two particle species. Since the total mass concentration is fixed the mass concentration $c_{p}$ of each species is determined by the adjusted mass fraction $M_{p}$ (see Eq. S19).

For Figure S2 where the adjusted mass fraction is ½ for both species this would mean a that the mass concentration $c_{p}$ both species is half that of the total mass concentration. This would explain why for cluster 0 the median of $U_{\mathrm{cross}}$ is similar for the total concentrations of 0.5 and 0.7 mg mL^–1^, because half the total mass concentration results in a mass concentration for the clusters of 0.25 and 0.35 mg mL^–1^ which is close to the velocity plateau found for the library data. For cluster 1 we found an increased $U_{\mathrm{cross}}$ compared to the same total mass concentrations in the library. This agrees with our expectations since the mass concentration for one species is smaller and thus crossing velocity should be higher. Accordingly, Figure S2 supports the proposal that library datasets with a similar concentration to the mass concentration of the clusters are a prerequisite for successful matching.

However, concentration alone cannot describe the complex behavior in the analyte. The correction using the mass concentration per species instead of the total mass concentration does not explain why for cluster 1 $U_{c\mathrm{ross}}$ at a concentration of 0.1 mg mL^–1^ is much larger than the $U_{\mathrm{cross}}$ for the single particle measurement of PSiO_2_ at $c_{\text{total}}$ of 0.05 mg mL^–1^. For cluster 0 we would expect a smaller $U_{\mathrm{cross}}$ for an adjusted mass concentration of 0.05 mg mL^–1^ as well due to the velocity plateau found in that region. Moreover, Cluster 1 shows a higher $U_{c\mathrm{ross}}$ at a total mass concentration of 0.5 mg mL^–1^ than expected from $c_{p}$. Only for a $c_{\mathrm{total}}$ of 0.7 mg mL^–1^ (i.e $c_{p}$ = 0.35 mg mL^–1^) cluster 1 fits into the library data. This observation leads us to the assumption of an interaction between porous and non-porous particles resulting in an increase of the crossing velocities at a concentration of 0.1 mg mL^–1^. At a concentration of 0.5 mg mL^–1^ this effect is still significant for porous particles but not for non-porous ones and for $c_{\mathrm{total}}$ of 0.7 mg mL^–1^ the interaction is negligible.


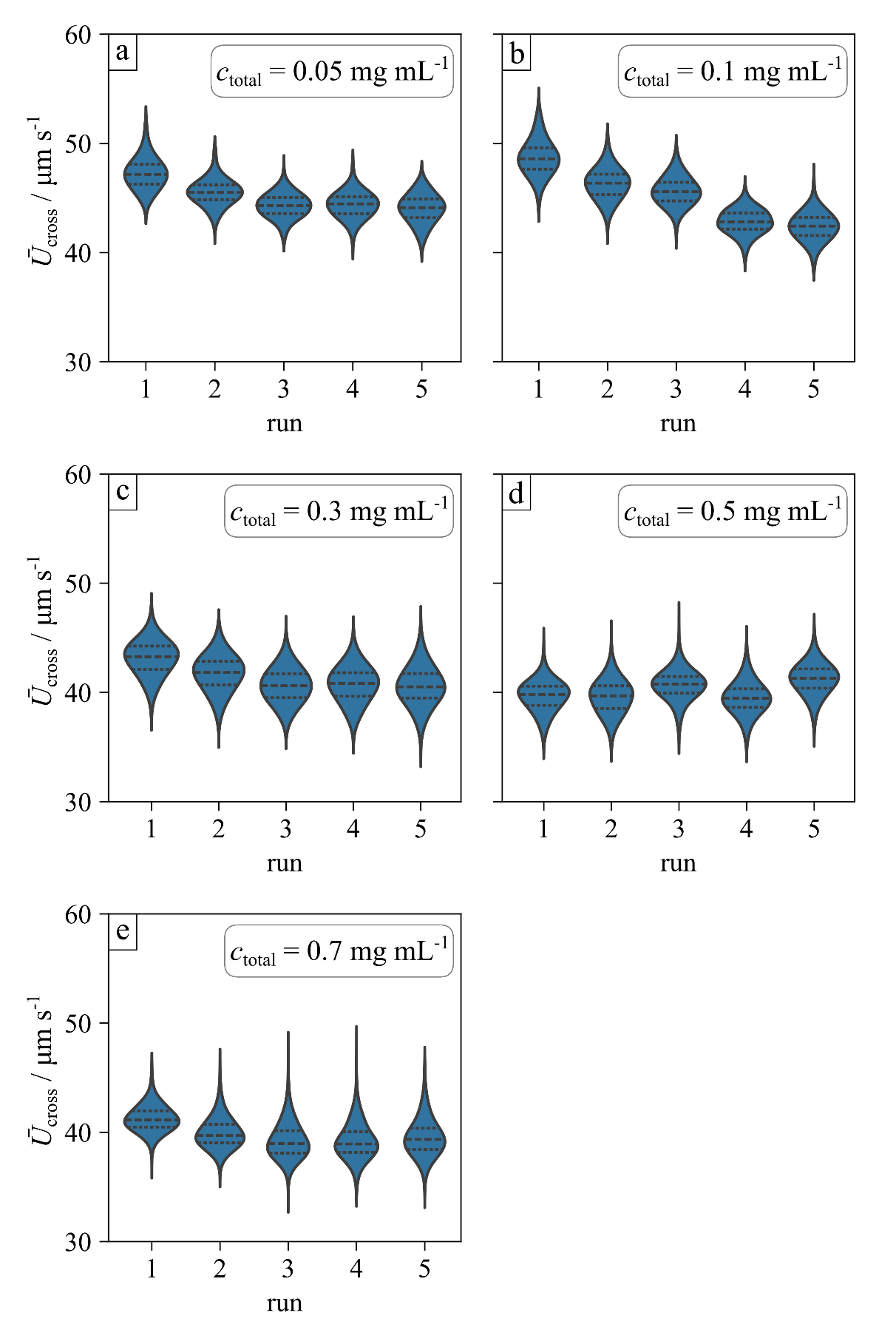


**Figure S11**. Illustration of particle distribution of porous silica particles (PSiO_2_) for 5 individual measurement runs under blue light illumination. Data collected at different particle concentrations: (a) *c* = 0.05 mg/mL, (b) *c* = 0.1 mg/mL, (c) *c* = 0.3 mg mL^–1^, (d) *c* = 0.5 mg mL^–1^, (e) *c* = 0.7 mg mL^–1^. Particle distribution is visualized using violin plots. Each successive run results in an increased cumulative light exposure of the sample. The particle velocity reduces with increasing run number but remains consistent and reproducible after the third experimental run. Therefore, data from the final three runs were merged and incorporated into the reference data library.


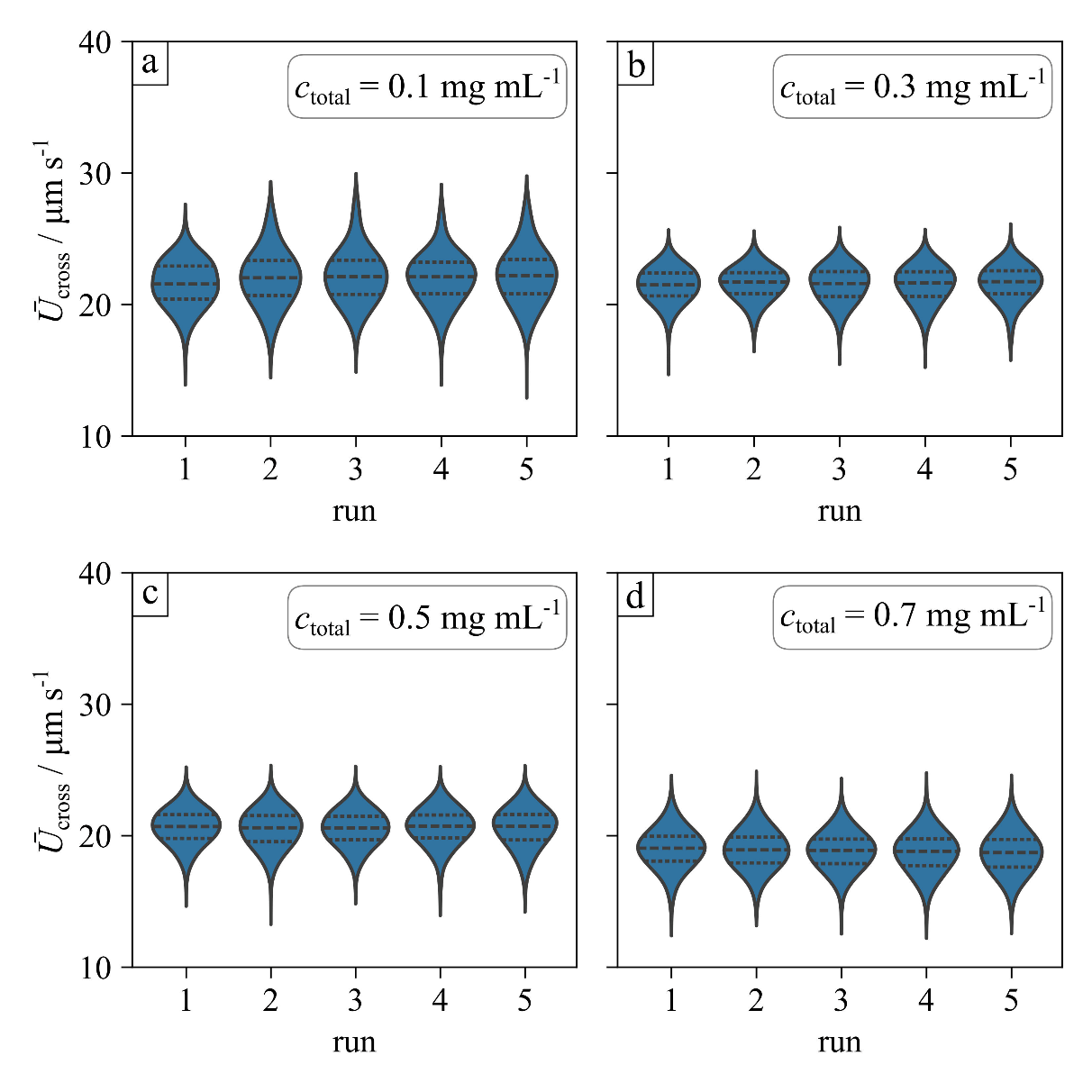


**Figure S12**. Illustration of particle distribution of non-porous silica particles (SiO_2_) for 5 individual measurement runs under blue light illumination. Data collected at different particle concentrations: (a) *c* = 0.1 mg mL^–1^, (b) *c* = 0.3 mg mL^–1^, (c) *c*= 0.5 mg mL^–1^, (d) *c* = 0.7 mg mL^–1^. Particle distribution is visualized using violin plots. Each successive run results in an increased cumulative light exposure of the sample. The particle velocity distribution remains statistically consistent and reproducible across all experimental runs, indicating high repeatability of the measurement conditions. Therefore, data from all runs were merged and incorporated into the reference data library.

# Pump type


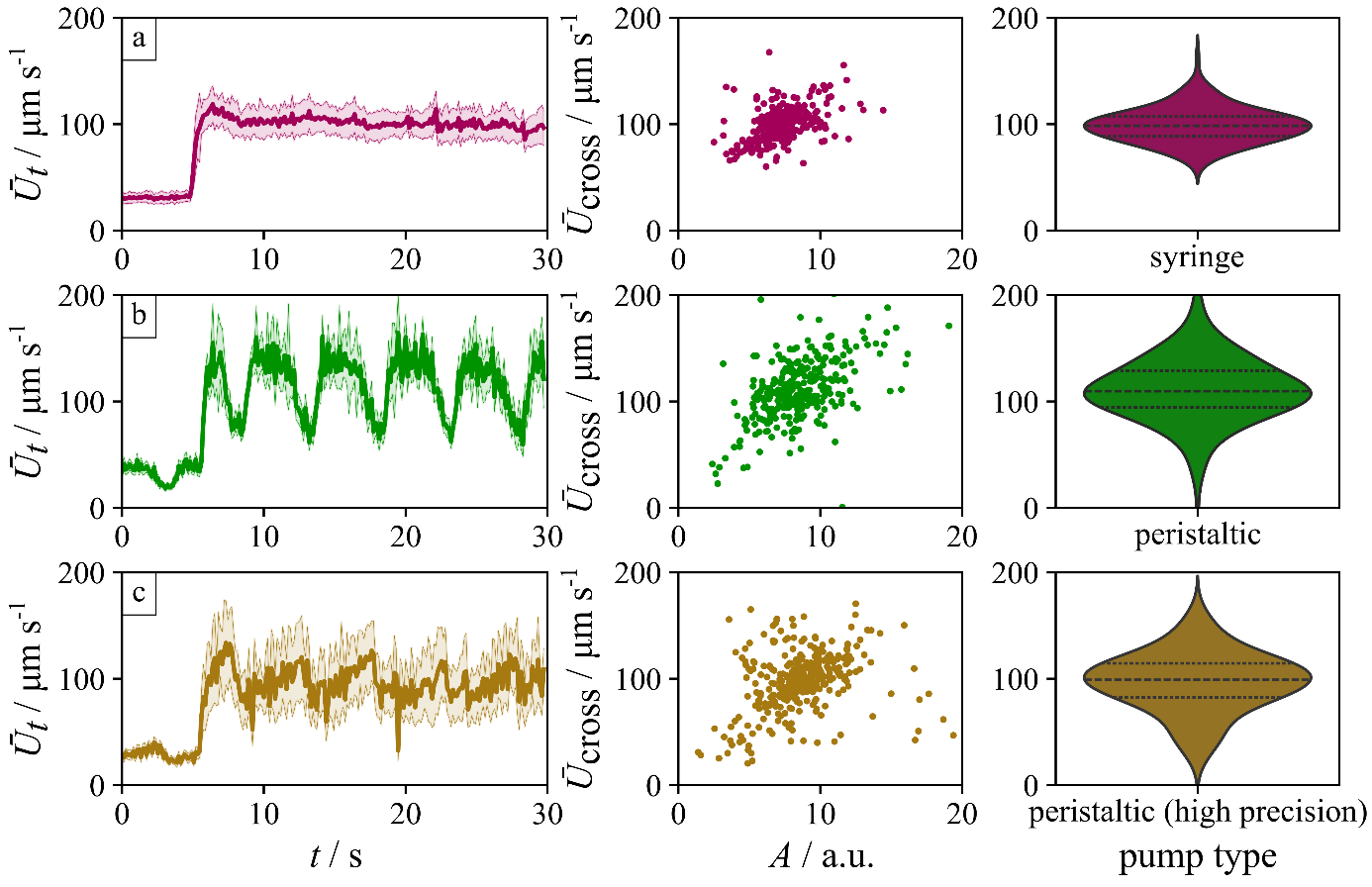


**Figure S13**. Validation of optimal pump type selection. (a) syringe pump, (b) peristaltic pump, (c) high-precision peristaltic pump. Data extracted from Video S5 are presented as follows: **(left)** temporal evolution of the average particle velocity; **(middle)** particle crossing velocity as a function of particle size; and **(right)** corresponding violin plots illustrating the distribution of crossing velocities. The blue-shaded region denotes the illumination interval (10–45 s), during which the data in the middle and right panels were exclusively computed.

# Image Acquisition time


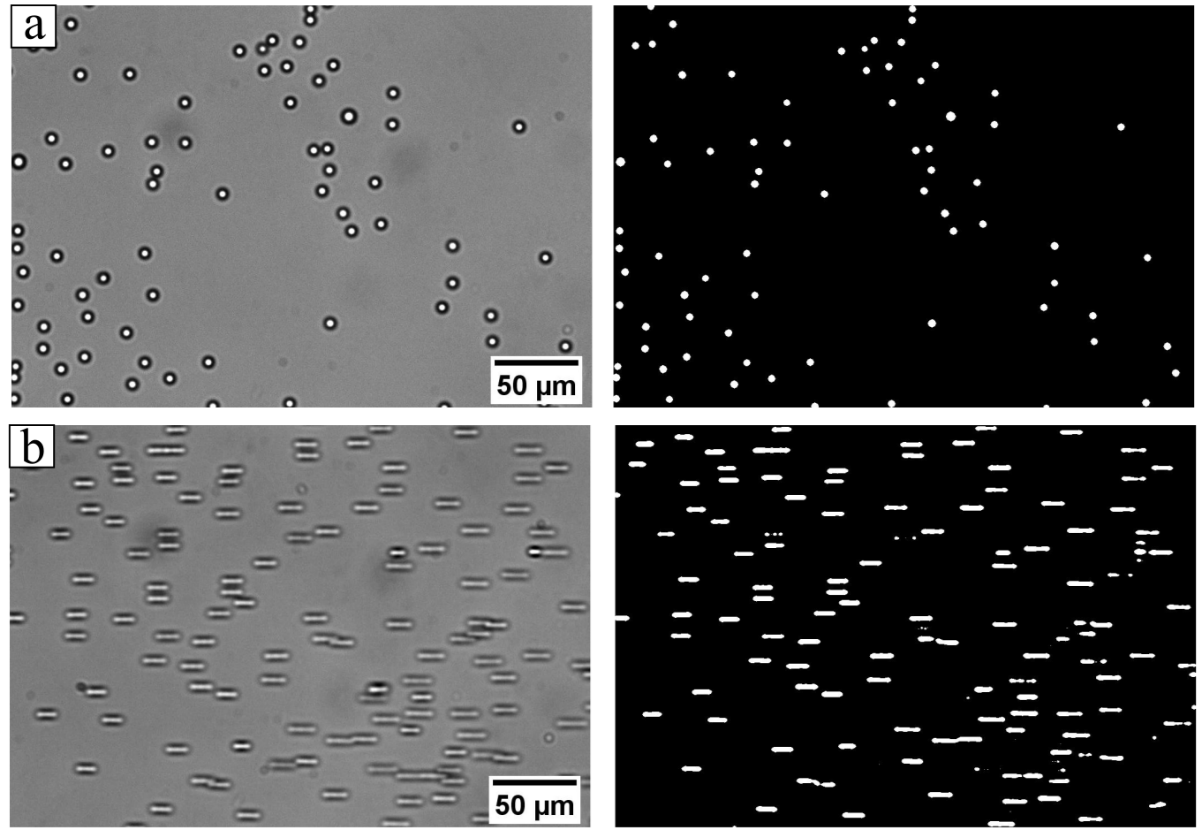


**Figure S14**. Effect of camera exposure time on particle velocity measurements. Displayed are snapshots under light illumination (**left**) raw data from VideoS5 and (**righ**t) in binary pixel information. Panels correspond to two exposure conditions: **(a)** short exposure time (1 ms) and **(b)** long exposure time (33 ms).


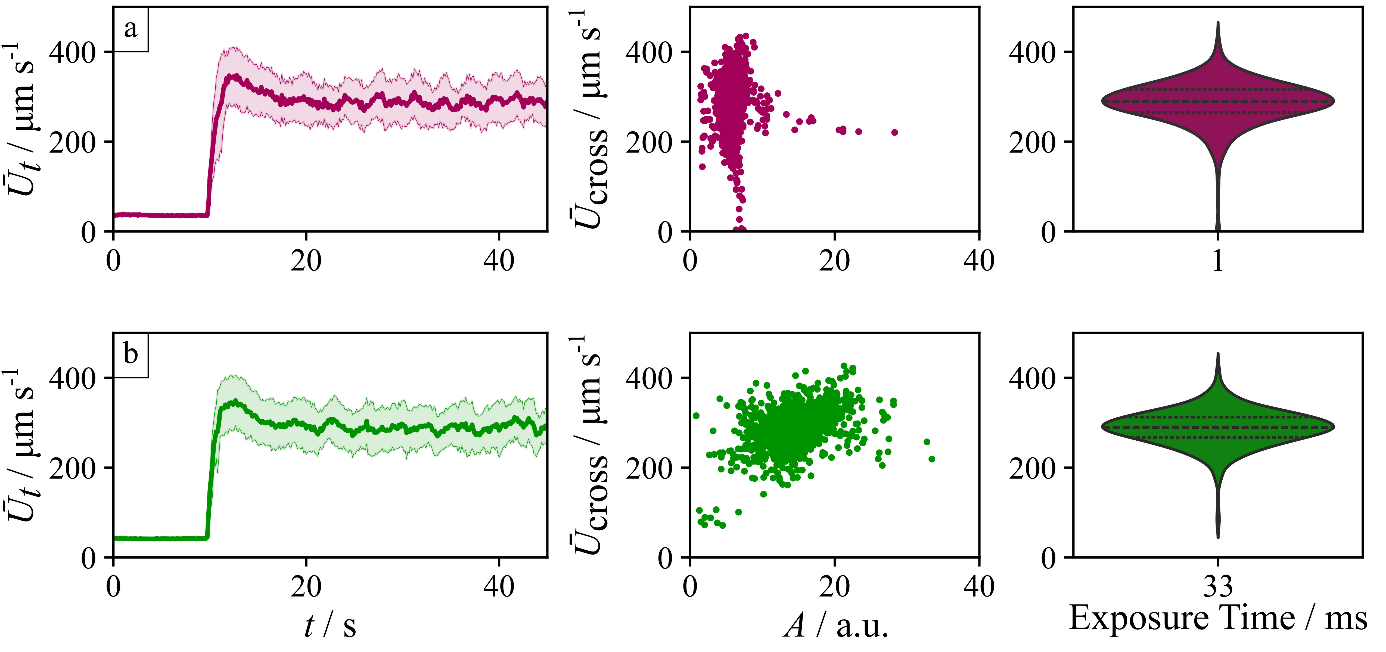


**Figure S15**. Effect of camera exposure time on particle velocity measurements, using data extracted from Video S6. Panels correspond to two exposure conditions: **(a)** short exposure time (1 ms) and **(b)** long exposure time (33 ms). For each condition, the following are shown: **(left)** average particle velocity as a function of time; **(middle)** clustered crossing velocity $U_{\text{cross}}$as a function of particle size $A$; **(right)** violin plots illustrating the distribution of $U_{\text{cross}}$corresponding to each exposure setting.

# Detailed Protocol for Sample Preparation

#
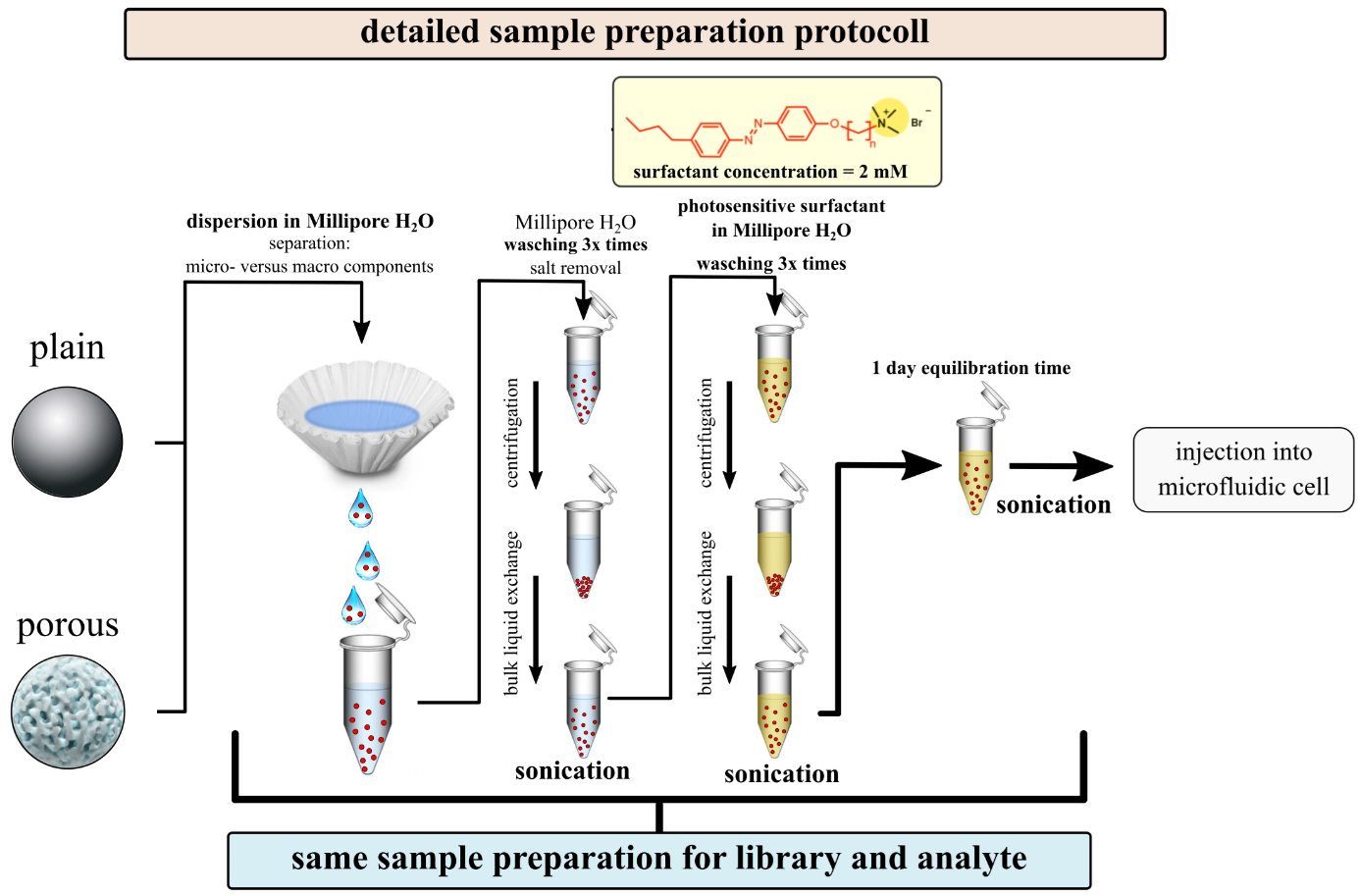


**Figure S16**. Detailed guidelines for sample preparation.

**More details regarding Sample Preparation Protocol for Colloidal Systems in Azobenzene-Based Surfactant Solutions**

Sample preparation should follow some important order. Here we give especially for readers who are not familiar with chemistry, chemical engineering and surface chemistry a small theoretical background for sample preparation together with chemical treatment.

**1. Impact of mm-sized structures - first step separation of macro sized objects:**

Big structures and impurities are faster than the analyte in micrometer scale, which cause during motion collision with particles, perturb its momentum, and compromise data fidelity, particularly in drift or motion based.

**Cleaning Principle:**

To minimize such interference, all dispersions should undergo **dead-end filtration** using an appropriate mesh size that excludes macroscopic objects while allowing colloidal microparticles (~1–10 µm) to pass. Avoid filter materials that may alter the solution's pH (e.g., certain cellulose-based papers). A polycarbonate or nylon membrane with a pore size much bigger than the analyte is typically sufficient.

**2. Removal of Dissolved Ionic Species: Importance of Salt-Free Conditions**

Already low ionic strength significantly impacts the light-induced local diffusioosmosis (*l*-LDDO) by decreasing the electrical double layer at the particle–fluid and substrate–fluid interface. Even small discrepancies in salt concentration between the colloidal analyte and the reference library can result in substantial deviations in light induced drift motion behavior.

**Cleaning Principle:**

To eliminate residual salt, the colloidal suspension should be thoroughly washed (≥3 cycles, see washing protocol) with high-purity deionized water (e.g., Millipore-grade). We strongly recommend using plastic (e.g., polypropylene) tubes for all sample handling and storage, as glass can leach ions that interfere with electrokinetic phenomena. Avoid prolonged contact with glassware during and after washing.

**3. Transfer into Azobenzene-Based Surfactant Solution**

The photoresponsive surfactant not only introduces light sensitivity but also acts as a traditional surfactant, capable of displacing loosely bound (physisorbed) contaminants from particle surfaces—analogous to conventional soap action. In addition, the cationic nature of the surfactant imparts a positive surface charge, which is essential for enhanced electrostatic stabilization.

The surfactant requires some time to be adsorbed, normally in the range of minutes, however, decreasing with increasing surfactant concentration. Also, the sample should be multiple times washed with the surfactant solution, to ensure the removal of physisorbed unwanted coatings and full coverage of photo-sensitive surfactants on the microparticles interface. Keep in mind that intentionally physisorbed coatings can be removed from the interface, too!

**Cleaning principle:**

We recommend washing the particles at least 3 times at desired concentration following the washing protocol. It is important to adjust the concentration between 1 to 3 mM, however to be consistent the concentration between library and analyte. A smaller concentration (~1 mM) will slightly enhance the strength of the l-LDDO but in trade off dynamic exchange of photo-sensitive surfactants over time, in other words the l-LDDO strength and light induced drift motion decreases very weakly over time. A higher concentration (2-3 mM) yields to slightly weaker magnitude of l-LDDO but into a more consistent dynamic exchange of photo-sensitive surfactants over time, thus into a longer stable light induced drift motion. If one does not know with which concentration to start, we recommend the surfactant concentration to set on 2 mM.

| **Concentration** | **Effect on l-LDDO** | **Surface Exchange Dynamics** |
| --- | --- | --- |
| **~1 mM** | Stronger l-LDDO; weaker long-term stability | Slower surfactant exchange; gradual decline in drift response |
| **2–3 mM** | Slightly reduced l-LDDO; better long-term stability | Faster surfactant exchange; consistent drift over time |

**4. Sample Storage and Equilibration**

After preparation, samples require an equilibration period to allow for full surfactant adsorption and interfacial stabilization. Light exposure can prematurely activate azobenzene isomerization, while elevated temperatures can further disrupt surface dynamics.

**Storage Guidelines:**

Store all prepared suspensions in dark conditions at ambient temperature for at least 24 hours prior to measurement. This resting period ensures equilibrium adsorption of the surfactant and helps achieve consistent particle velocities across different samples (e.g., between library and analyte), improving reproducibility.

**5. General washing procedures**

**Centrifugation and supernatant exchange:** Sediment the particles via centrifugation. Gentle g-force depending on particle size and density, typically 500 RPM, for 5minutes. Use small slowdown rate. Then carefully decant and discard the supernatant. This step removes excess unbound surfactant and soluble impurities.

**Point-by-point Washing Protocol**

1. **Initial Dispersion**: Disperse the microparticles in either ultrapure (Millipore-grade) water or a pre-prepared aqueous surfactant solution. Homogenize the suspension by sonication for 1 minute to break up aggregates and ensure uniform dispersion.
2. **Equilibration Phase**: Allow the particle suspension to equilibrate for a minimum of 5 minutes to stabilize particle–solvent and particle–surfactant interactions.
3. **Centrifugation and Supernatant Exchange**: Sediment the particles via centrifugation. Gentle g-force depending on particle size and density, typically 500 RPM, for 5minutes. Use small slowdown rate. Then carefully decant and discard the supernatant. This step removes excess unbound surfactant and soluble impurities.
4. **Re-dispersion**: Immediately re-disperse the pelleted particles in fresh solvent to prevent drying or aggregation. Continuous wetting of the particles throughout the process is crucial to maintaining colloidal stability and preventing irreversible adhesion.
5. **Final Transfer and Equilibration**: Transfer the particles into the final aqueous photosensitive surfactant solution at the desired concentration. In case with the photosensitive surfactant, disperse the sample with Millipore, and finally dilute with the surfactant stock solution concentration. Choose a surfactant total concentration between 1-3 mM, not lower or higher. Store the suspension under dark, ambient-temperature conditions for extended equilibration (typically 24 hours) prior to analysis. Light and thermal exposure should be minimized during this phase to prevent premature isomerization of the azobenzene moiety

# Careful points to consider

***S12.1 Careful points to consider – sample preparation***

It is critical to ensure precise and consistent mixing of the particles with the surfactant solution, in alignment with both the library and analyte measurements. This is because the phoretic mobility of the particles, along with their light-induced drift motion, is highly sensitive to the surfactant concentration, the ionic strength of the solution, and the specific sample preparation method. Therefore, maintaining strict consistency in sample preparation for both the library and analyte is essential. Even slight deviations in absolute velocity can lead to significant discrepancies in data interpretation when comparing data clusters.

For sample preparation microparticles must be transferred into an aqueous solution containing the azobenzene-based surfactant.  We recommend initially washing the particles with Millipore water, followed by the addition of the surfactant solution to achieve the desired final concentration. A detailed schematic of the microparticle washing cycle is provided in **Figure S14** (Supporting Information), illustrating the following steps:

(I) Disperse the particles in the solvent (water or aqueous surfactant solution) by sonicating them for 1 minute.

(II) Allow the particles to equilibrate in the solvent for at least 5 minutes.

(III) Remove/replace the supernatant by first centrifugation with subsequent removal of the supernatant

(IV) Immediately re-disperse the particles, ensuring they remain wet throughout the process.

(V) After transferring the particles to the photosensitive surfactant solution, store them for further equilibration (e.g., 1 day), avoiding exposure to light or heat during this period until the final analysis.

The examples described above, along with the schematics in **Figure 5** and the detailed protocol in **Figure S16** and **Section S11** (Supporting Information), serve as guidelines. The number of washing cycles and equilibration times may be adjusted, but must remain consistent across all measurements, for both the library and analyte. Inconsistencies in preparation can result in mismatched light-induced velocities and lead to misidentification. As a rule of thumb, we recommend three washing cycles with Millipore water and the photosensitive surfactant solution.

***S12.2 Careful points to consider – Outlier removal***

As mentioned in section 2.3 outliers in the data must be removed before the clustering algorithm is applied. This is necessary since they result from mis-tracking, impurity of the sample and particle clusters. In these cases, a matching with the library is impossible (mis-tracking) or not yet possible (impurity of the sample and particle clusters). Moreover, the outliers form their own clusters in the data, resulting in more than two clusters which is the number we would expect from two species. This could worsen the performance of the clustering analysis and thus must be avoided.

We identified four types of outliers depicted in **Figure 6** and **Video S4**: Colliding particles (Figure 6a,b, **Video S4**, Panel a,b), particles with a diameter much larger than the average (Figure 6d, **Video S4 Panel d**), in and out moving particles (Figure 6e, **Video S4**, Panel e) and particle clusters (Figure 6f, **Video S4**, Panel f).

The colliding of two or more particles normally results in the deflection of the particles from their path along the streamline of the fluid. This allows faster particles to outrun slower particles in their direction of movement. On its way around the slower particle the faster particle is accelerated. On its own this would not cause outliers to be two or three times faster than the average since it is only a small perturbation in its path. These outliers can be explained by mis tracking by the utilized software. Tracks are lost when the displacement from one to the next frame are large in the direction orthogonal to the streamlines, which is the case when particles are deflected. Thus, the path of the particle gets fragmented in several tracks during the process of outrunning. This results in many outliers which are much faster than the average particles but have the same average diameter, see **Figure 6c** violet oval marker. Luckily this issue is also the key to outlier identification and removal. The resulting short path length of the tracks serves as an identifier, and we removed all tracks which’s path length is smaller or equal to that of the 0.15 Quantile of the tracked path lengths. Another sign for a collision is the acceleration or deacceleration of the particles involved. This change in the velocity leads to an increase in the standard deviation of the velocity. Therefore, removed all with standard deviation larger or equal to the 0.9 quantile of all standard deviations of particle velocities.

While studying the second type of outliers we found that the majority of clusters consisted of two particles. The outliers originated from clusters with one particle of each material. Accordingly, the cluster contains one particle which is more phoretically active and thus can move faster than the other one. Since they stick together this results in a less active particle which moves at the same velocity as the more active particles. Moreover, the dragging particle gets slower since the mass of the cluster is larger but the contribution of the attached particle to the activity of the cluster is negligible. At the end this results in two outliers, one where area *A* is the one expected for the less active particle, but the velocity corresponds to the stronger active particle and other one where more active particles are considerably slower than the average. These outliers are marked with green oval in **Figure 6c**. They are much harder to identify and are therefore treated individually for each measurement and removed manually. This problem could be solved in the future by using an image to object AI application recognizing the clusters and treating them individually.

Another source of outliers are particles that are captured at the borders parallel to the streamlines of the micrographs. Since the tracking software only captures the part which is inside the image their area is smaller than the average particle (see **Figure 6e**). This results in tracks that record the expected velocity but with a small particle area. These outliers are marked with orange oval in Figure 6c. Moreover, particles at the edge offend exit and re-enter the observed area. This results in an un-continuous tracking of the path of these particles. Accordingly, we can treat these outliers like those produced by colliding particles by removing tracks with short path length.

The last type of outliers we found were particles with a much larger radius than the average radius. Their larger cross-sectional area results in a velocity larger than the average (see **Figure 6c** cyan oval). They have been treated by removing all tracks with a particle area *A* larger or equal to that of the 0.99 Quantile.

***S12.3 Careful points to consider – Library design***

The core of the particle identification relies on the comparison between the quantiles of *U*_cross_ from the library and the analyte data. To maximize identification accuracy from quantile comparison, it is essential to achieve a close match between crossing velocities *U*_cross_ of both datasets.

In general, the library must be constructed using the same measurement conditions as the analyte, with the key difference being that the physical (e.g., size, shape) and chemical (e.g., material, surface functionalization, porosity) properties of the particles in the library are well known and clearly classified. Data acquisition for the library involves recording trajectories of the particles following the flow streamlines under light illumination at fixed wavelength and intensity ideally for one particle species at a time and importantly, across a range of particle concentrations. This variation is crucial for covering the expected concentration range of the analyte in bi-particle mixtures. To better understand the design of the library and the core parameters used for classification, we briefly explain the physical phenomenon behind the LDV dependence (~*U*_cross_ change as recording parameter) on parameters such as flow rate, particle size, particle surface chemistry,^[^^[[4]](#endnote-4)]^ intensity,^[^^[[5]](#endnote-5)]^ wavelength and recently reported also on the particle concentration.^[^^[[6]](#endnote-6)]^

*Flow rate, intensity, wavelength*: Light intensity and wavelength influence the dynamic exchange of the photo-sensitive surfactant isomer,^[Ref 16 main article]^ which in turn affect the strength of particle activity.^[Ref 17 main article]^ This activity determines the lift-off behavior, while the shear rate *S* (dependent on flow rate and cell geometry) modulates the magnitude of LDV for all particles equally. In practice, flow rate, intensity and wavelength can be precisely controlled, and are kept constant across both library and analyte measurements.  As a result, only minimal variation in *U* is expected between recordings.

*Particle size*: Particle size has a strong influence on the magnitude of *U*, larger particles generally exhibit higher velocities. This applies to both fully sedimented and lifted particles under light illumination.^[5]^ Thus, it is important to classify *U*_cross_ in the library according to particle size at a given surface chemistry.  For demonstration purposes, we used a system with particles of uniform size (5 µm diameter) and varied only their surface area by comparing plain and porous particles.

*Particle surface chemistry*: The strength of light-induced activity is highly sensitive to interfacial properties, including surface functionalization, bulk material, surface area, and surface charge. These factors influence the surfactant’s storage capacity and exchange dynamics upon illumination. For further details we refer the reader to relevant literature.^[4,5,6]^ Consequently,  it is critical to classify the library by interfacial properties such as surface area (e.g., plain vs. porous), surface functional groups (e.g., –OH, –NH₂, –NR₃⁺, –C₁₈), and particle material (e.g., silica, polystyrene, polymethacrylic acid).

In our demonstration, we intentionally fixed most surface chemistry parameters. Both particles shared the same particle bulk material (silica) and surface functionalization (–OH). The only variation was the effective surface area, allowing us to classify particles based solely on porosity type–i.e., plain versus porous surface.

*Particle concentration*: We recently demonstrated that the strength of particle activity depends on the distance on the nearest neighboring active particle. This is because adjacent particles generate spatial *cis*-isomer gradients, which, when overlapping, reduce the net activity of each particle. As particle concentration increases, the average interparticle distance decreases, leading to stronger gradient overlap and, consequently, a reduction in activity. This results in a lower levitation tendency and a corresponding decrease in *U*_cross_.^[6]^

In practice, natural analyte samples rarely exhibit uniform particle concentrations neither in absolute terms nor in the relative proportions of different particle types. This is important because the activity of one colloid is influenced by the neighboring colloids.^[6]^ Assuming a bi-particle mixture contains both strongly and weakly active colloids, their mutual repulsion via *l*-LDDO depends on their relative positions and activity strengths. Weak–weak pairs can approach closely before repulsion sets in, while strong–strong pairs maintain greater distances. Mixed strong–weak pairs align somewhere in between. While simplified, this model illustrates the complex collective behavior: the closer the particles (i.e., the higher the concentration), the more their activity is influenced by neighboring particles. The effective strength of *l*-LDDO and the resulting LDV both decrease with increasing particle concentration under fixed illumination.^[6]^

This concentration-dependent behavior causes deviations in LDM between single dispersions recorded in the library and bi-particle mixtures in the analyte, even at the same total particle concentration. To minimize such discrepancies, the library must be designed to match the individual particle concentrations of the analyte, not just the total concentration. We propose a simple technical solution to address this issue.

If we assume, that only weak-weak or strong-strong interactions dominate active particles are interacting neglecting interactions between weak and strong active particles then the relevant factor for LDV (~*U*_cross_​) is the absolute concentration of each particle species. For an analyte composed of two finite particle types, labelled Cluster 0 and 1, the total particle concentration *c*_tot,analyte_ is:

| $c_{tot,analyte}=c_{0,\mathrm{analyte}}+c_{1,\mathrm{analyte}}$ | Eq. S29 |
| --- | --- |

where *c*_0_​ and *c*_1_​ are the concentrations of each particle type. is a simple sum from ensembles of Cluster 0 and 1 with corresponding particle concentration *c*_0_ and *c*_1_, where the index 0 and 1 represents the particle type in the analyte. For the library, where *U*_cross_ of from particle mixture only a single dispersion of particles with well-known surface properties is measured, the total concentration *c*_tot,library_ is:

| $c_{tot,library}=c_{X,library}$ | Eq. S30 |
| --- | --- |

with *X*=0 or 1 representing the particle type. The LDV values (~*U*_cross_) are only comparable if the condition *c*_0,library_~ *c*_0,analyte_ and *c*_1,library_~ *c*_1,analyte_ is satisfied. Since the ratio of Cluster 0 to Cluster 1 may vary between analyte samples, the corresponding concentrations *c*_0,analyte_ and *c*_1,analyte_ must also vary. Therefore, it is essential to compare the LDV values of the clusters with datasets from the library which total concentration is similar to the absolute concentration of the species in the mixture, *c*_x,library_~ *c*_x,analyte_, rather than compare library and analyte by the total concentration. At equal total concentrations, particles in a mixture may exhibit faster LDV than in the library because the concentration of each species is lower, i.e., more diluted, resulting in fewer particle–particle interactions and stronger individual activity. This leads to stronger levitation and faster LDV in the analyte compared to the library. Experimental data in **Figure S10** and **Section S8** (Supporting Information) support this, showing that *U*_cross_​ values from the analyte only match those from the library when the library data is taken at a lower particle concentration.

Since analyte samples may vary in both total concentration and particle composition, iterating the library across a range of particle concentrations is critical for accurate *U*_cross​_ matching. This is one of the most important considerations in library design. Principles are illustrated in **Figure S10**. By considering that we measured the value of *U*_cross_ for every single dispersion, i.e. plain and porous silica colloids in a concentration from *c*_p_ = 0.1–0.7 mg/ml, where the data is plotted for both particles in **Figure S12**. Every library data at one fixed concentration contains 5 individual measurements of 30 seconds recording time. Data in **Figure S11** exhibits that the last 3 out of 5 data sets show a constant *U*_cross_. These are combined together and represent the library data set at one particle concentration. The same iterations are done with all other concentrations.

***S12.4 Careful points to consider – Pump type***

A critical parameter that must be precisely controlled for reliable material identification using the LDM is the type of pumping system employed and the resulting flow rate stability. Accurate and repeatable delivery of a constant flow rate, especially over short, discrete time intervals on the order of milliseconds, is essential to ensure consistent hydrodynamic conditions within the measurement system. In this work, we utilized a high-precision syringe pump capable of delivering fluid at low, steady flow rates with minimal pulsation and noise. The syringe pump enables fine control over volumetric flow rate, ensuring that a constant pressure gradient is maintained throughout the flow path, which is crucial for particle motion uniformity and accurate motion analysis. While alternative pump types can be employed, it is important to avoid pumps that introduce periodic pressure fluctuations. Pumps such as peristaltic or gear pumps inherently produce pulsatile flow profiles due to their mechanical operation. These pulsations manifest as transient accelerations and decelerations in particle motion, leading to flow instabilities and measurement artifacts in LDM-based classification.

To demonstrate the significance of pump-induced flow characteristics, we present a comparative study in **Video S5**, where three different pumps were evaluated displayed in **Figure S13**: a syringe pump and two commercially available peristaltic pumps from different manufacturers. Similar as previously analyzed the objects and the motion is tracked via trajectory analysis, then calculated as (1) time-revolved average velocity, (2) *U*_cross_ classified per object size and displaying velocity probability distribution via (3) violin plot analysis.

The results in **Figure S13** indicate that although the long-term average flow rates of all three systems converge, the instantaneous velocity profiles differ significantly. The peristaltic pumps exhibit periodic peaks and troughs in velocity, directly reflecting the pulsation pattern of the pump mechanism. These velocity oscillations cause non-uniform particle motion and may lead to broad, overlapping data clusters in both *U*_cross_ classification and velocity distribution plots.

Such scattering minimizes the accuracy of material classification, particularly when distinguishing between multiple particle fractions. Overlapping clusters hinder precise discrimination, leading to misclassification or the inability to resolve distinct particle populations (~ distinct data clusters). Moreover, during peak flow intervals, particle velocities exceed the temporal resolution of the imaging system, causing motion blur and longitudinal smearing in the recorded video. As a result, particle recognition algorithms may interpret identical particles as different objects based on apparent size differences, further amplifying data dispersion and generating erroneous outliers.

Conversely, the syringe pump with its low flow-rate noise and smooth pressure delivery yields compact, well-separated data clusters. This ensures a high grade of reliable material classification of particle populations. For optimal performance, we strongly recommend the use of high-precision pumps that offer excellent flow stability and minimal pulsation. Furthermore, it is essential to ensure that the fluid reservoir, tubing, and pump components are uniformly filled with the aqueous solution containing the photo-sensitive surfactants, to avoid unwanted dilution in the microfluidic chamber.

***S12.5 Careful points to consider – Image acquisition time***

One critical point for accurate data interpretation is how data have been recorded via video microscopy. This also includes image acquisition, as too long exposure time for fast moving particles may yield into smeared particles in longitudinal flow direction. Subsequent object analysis than reveal wrongly measured sizes for particles and as such data cluster are miss classified, compared with the wrong size of particles and consequently misinterpreted by the software.

To minimize motion blur and improve temporal resolution in high-speed video analysis, the exposure time must be reduced as much as practical. To quantify the impact of exposure duration, we conducted image acquisition at a constant frame rate of 30 FPS under two distinct exposure conditions: 33 ms and 1 ms per frame. All other acquisition parameters were held constant, with the exception of the illumination intensity of a red LED light source (*λ* = 625 nm). For the short exposure condition (1 ms), the luminous flux of the red light was significantly increased to compensate for reduced photon integration time, thereby maintaining consistent image brightness across both exposure settings.

A demonstration is shown in **Video S6**, where porous microparticles are illuminated with UV light (*λ* = 365 nm) to provide the maximum boost velocity ^[^^[[7]](#endnote-7)]^ from the fastest photoisomerization kinetics at given wavelength.^[^^[[8]](#endnote-8),^^[[9]](#endnote-9)]^ Data shows in **Figure S14** snapshots in dark and under light illumination classified by exposure time for the real recorded image and calculated binary pixel information (~thresholded image). It is evident from **Video S6** and **Figure S15a,b** that prolonged exposure times lead to motion blur artifacts, manifesting as smeared and elongated particle shapes along the direction of motion. In contrast, at reduced exposure durations, the particles consistently appear spherical, accurately reflecting their true geometry. This effect is particularly pronounced under illumination (activity on), where longer integration times results in image elongation due to the convolution of fast particle motion and exposure duration. Importantly, despite these shape distortions, the measured particle velocities remain unaffected by the choice of exposure time. This is demonstrated in **Figure S15**, where time-resolved average velocities are nearly identical for both exposure settings. This consistency is expected, as particles traverse the same distance between consecutive frames due to the fixed frame rate (30 FPS), regardless of exposure time. However, the pixel area calculated are at long exposure significantly bigger, revealing particles with a clear aspect ratio as an artifact.

This causes in the plot’s velocity *U*_cross_ against the particle area a shifted data cluster or even a broadened one. Thus, Cluster classification might be affected revealing a different particle size as artifact than physically present. Consequently, this introduces bias in data interpretation, as particles are misclassified or compared based on size dimensions not reflecting their true size. And since *U* scales linear with the particle area the measured velocity would be than classified "slower".

Accurate size determination of spherical particles regardless of their motion velocity within a single recording sequence is achieved by employing a short exposure duration of 1 ms, even though the inter-frame interval remains fixed at 33 ms (30 FPS). This short exposure minimizes motion blur, enabling reliable morphological analysis of both slow- and fast-moving particles within the same dataset.

Moreover, reducing the exposure time mitigates the risk of frame loss or temporal artifacts due to pixel-level readout and signal clearing delays inherent to the image sensor. Since each pixel requires a finite recovery time (i.e., electronic reset or "reboot") following exposure, longer exposure durations can cause dead-time effects, potentially leading to missed or corrupted frames. It is also important to note that the red LED illumination used (*λ* = 625 nm) does not induce photo-isomerization in the sample system. Therefore, the intensity of the red light can be increased without perturbing the photochemical properties of the sample. This allows for high-intensity illumination to compensate for the reduced photon collection time at short exposures, ensuring optimal image brightness and signal-to-noise ratio.

# Microscope Setup


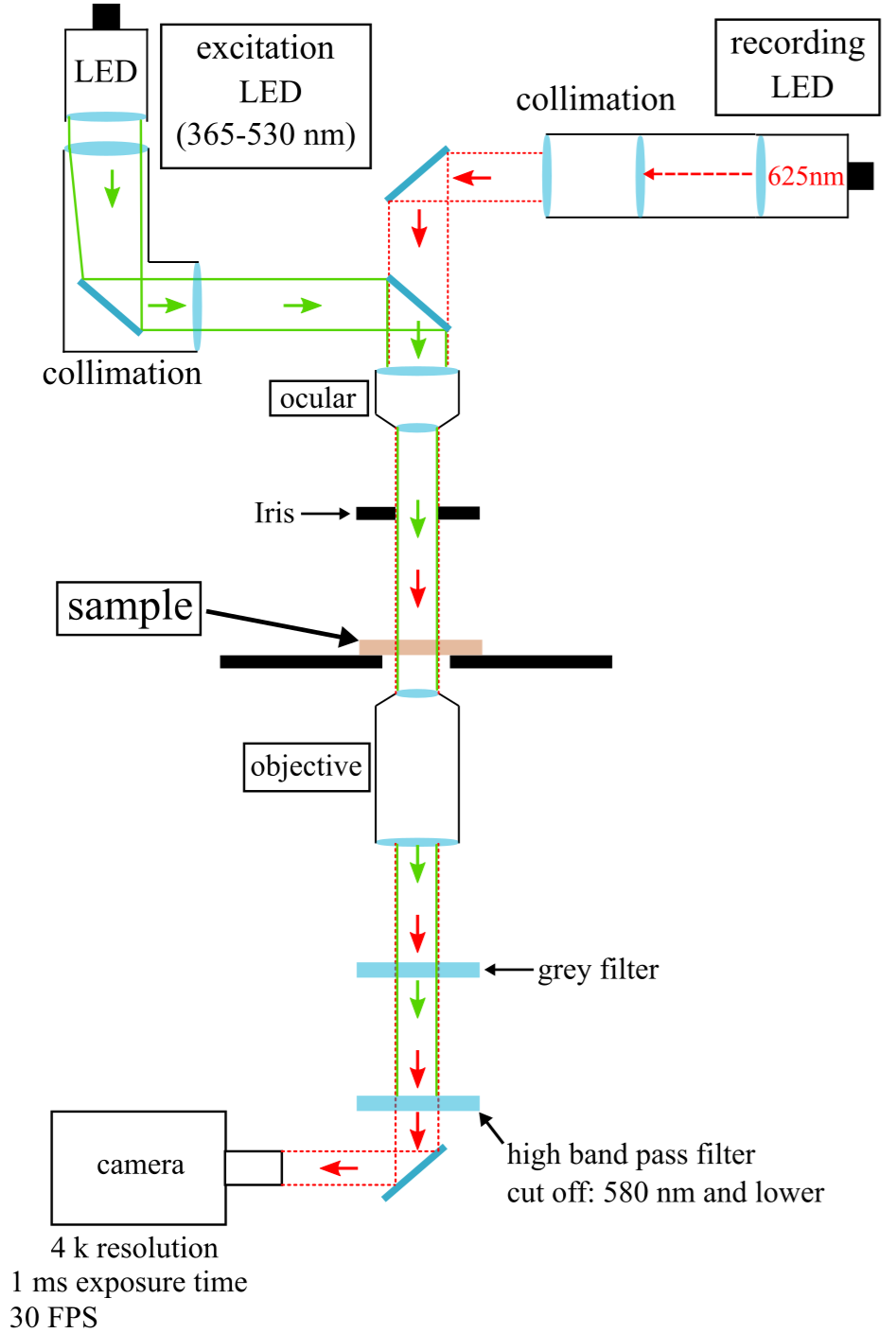


**Figure S17**. Schematic representation of the microscope setup.

# References for Supporting Information

1. [] Raschka, Sebastian, Yuxi Hayden Liu, and Vahid Mirjalili. *Machine Learning with PyTorch and Scikit-Learn: Develop machine learning and deep learning models with Python*. Packt Publishing Ltd, 2022. [↑](#endnote-ref-1)
2. []Gaussian Mixture Model. geeksforgeeks.org. Retrieved 04:19p.m., July 31, 2025, from <https://www.geeksforgeeks.org/machine-learning/gaussian-mixture-model/> [↑](#endnote-ref-2)
3. [] Gaussian Mixture Model. *Brilliant.org*. Retrieved 11:21, July 31, 2025, from <https://brilliant.org/wiki/gaussian-mixture-model/> [↑](#endnote-ref-3)
4. [] Rohne, F.; Vasquez-Muñoz, D.; Santer, S.; Bekir, M. Improving the surface chemistry-based separation of microparticles – a modified gravitational field flow fractionation and what key parameters are essential, *J. Chromatogr. A*., **2025**, *1762*, 466368. [↑](#endnote-ref-4)
5. [] Vasquez-Muñoz, D.; Popescu, M. N.; Sharma, A.; Rohne, F.; Meier, I.; Ortner, P; Loebner, S.; Benson, J. R.; Lomadze, N.; Eickelmann, S. Santer, S.; Bekir, M. Surface-Sensitive Fractioning of Flowing Colloidal Suspensions Sedimented at a Photochemically Active Wall, *Small*, **2025**, *21*, 2500012. <https://doi.org/10.1002/smll.202500012> [↑](#endnote-ref-5)
6. [] Rohne, F.; Vasquez Muñoz, D.; Meier, I.; Lomadze, N.; Santer, S.; Bekir, M. Statistical distribution of elevation from a planar interface of phoretically active microparticles, Lab Chip **2025**, *25*, 4106-4118 <https://doi.org/10.1039/D4LC01092B> [↑](#endnote-ref-6)
7. [] Vasquez Muñoz, D.; Rohne, F.; Meier, I.; Sharma, A.; Lomadze, N.; Santer, S.; Bekir, M., Light Induced Material Motion Fingerprint – A Tool Towards Selective Interfacial Sensitive Fractioning of Microparticles via Microfluidic Methods, *Small* **2024**, *20*, 202403546. <https://doi.org/10.1002/smll.202403546> [↑](#endnote-ref-7)
8. [] Titov, E.; Sharma, A.; Lomadze, N.; Saalfrank, P.; Santer, S.; Bekir, M. Photoisomerization of Azobenzene-Containing Surfactant within a Micelle, *ChemPhotoChem* **2021**, *5*, 926. <https://doi.org/10.1002/cptc.202100103> [↑](#endnote-ref-8)
9. [] Sharma, A.; Bekir, M.; Lomadze, N.; Santer, S. Photo-Isomerization Kinetics of Azobenzene Containing Surfactant Conjugated with Polyelectrolyte, *Molecules* **2021**, *26*, 19. <https://doi.org/10.3390/molecules26010019> [↑](#endnote-ref-9)
